# Inhibition of fatty acid desaturation is detrimental to cancer cell survival in metabolically compromised environments

**DOI:** 10.1186/s40170-016-0146-8

**Published:** 2016-04-01

**Authors:** Barrie Peck, Zachary T. Schug, Qifeng Zhang, Beatrice Dankworth, Dylan T. Jones, Elizabeth Smethurst, Rachana Patel, Susan Mason, Ming Jiang, Rebecca Saunders, Michael Howell, Richard Mitter, Bradley Spencer-Dene, Gordon Stamp, Lynn McGarry, Daniel James, Emma Shanks, Eric O. Aboagye, Susan E. Critchlow, Hing Y. Leung, Adrian L. Harris, Michael J. O. Wakelam, Eyal Gottlieb, Almut Schulze

**Affiliations:** Gene Expression Analysis Laboratory, Cancer Research UK London Research Institute, 44 Lincoln’s Inn Fields, London, WC2A 3LY UK; Cancer Research UK, Beatson Institute, Switchback Rd, Glasgow, G61 1BD UK; Babraham Institute, Babraham Research Campus, Cambridge, CB22 3AT UK; Department for Biochemistry and Molecular Biology, Theodor-Boveri-Institute, University of Würzburg, Am Hubland, 97074 Würzburg, Germany; Molecular Oncology Laboratories, Weatherall Institute of Molecular Medicine, University of Oxford, John Radcliffe Hospital, Oxford, OX3 9DS UK; High Throughput Screening Facility, The Francis Crick Institute, Lincoln`s Inn Fields Laboratories, 44 Lincoln`s Inn Fields, London, WC2A 3LY UK; Bioinformatics and Biostatistics Service, The Francis Crick Institute, Lincoln`s Inn Fields Laboratories, 44 Lincoln`s Inn Fields, London, WC2A 3LY UK; Experimental Histopathology, The Francis Crick Institute, Lincoln`s Inn Fields Laboratories, 44 Lincoln`s Inn Fields, London, WC2A 3LY UK; Department of Surgery and Cancer, Imperial College London, Hammersmith Hospital, Du Cane Road, London, W12 0NN UK; AstraZeneca, Mereside, Alderley Park, Macclesfield, SK10 4TG UK; Comprehensive Cancer Center Mainfranken, Josef-Schneider-Str. 6, 97080 Würzburg, Germany; Present address: The Breakthrough Breast Cancer Research Centre, The Institute of Cancer Research, London, SW3 6JB UK

**Keywords:** Lipid desaturation, Breast cancer, Prostate cancer, Lipidomics, SCD

## Abstract

**Background:**

Enhanced macromolecule biosynthesis is integral to growth and proliferation of cancer cells. Lipid biosynthesis has been predicted to be an essential process in cancer cells. However, it is unclear which enzymes within this pathway offer the best selectivity for cancer cells and could be suitable therapeutic targets.

**Results:**

Using functional genomics, we identified stearoyl-CoA desaturase (SCD), an enzyme that controls synthesis of unsaturated fatty acids, as essential in breast and prostate cancer cells. SCD inhibition altered cellular lipid composition and impeded cell viability in the absence of exogenous lipids. SCD inhibition also altered cardiolipin composition, leading to the release of cytochrome C and induction of apoptosis. Furthermore, SCD was required for the generation of poly-unsaturated lipids in cancer cells grown in spheroid cultures, which resemble those found in tumour tissue. We also found that SCD mRNA and protein expression is elevated in human breast cancers and predicts poor survival in high-grade tumours. Finally, silencing of SCD in prostate orthografts efficiently blocked tumour growth and significantly increased animal survival.

**Conclusions:**

Our data implicate lipid desaturation as an essential process for cancer cell survival and suggest that targeting SCD could efficiently limit tumour expansion, especially under the metabolically compromised conditions of the tumour microenvironment.

**Electronic supplementary material:**

The online version of this article (doi:10.1186/s40170-016-0146-8) contains supplementary material, which is available to authorized users.

## Background

Altered metabolism has now been established as a central hallmark of oncogenic transformation [1]. Aberrant activation of oncogenic signalling pathways and loss of tumour suppressor function alters metabolic processes in cancer cells to satisfy their increased energetic and biosynthetic demand [2, 3]. Moreover, the reduced availability of nutrients and oxygen in poorly vascularised regions of solid tumours also affects the metabolic activity of cancer cells [2, 4]. This metabolic reprogramming supports cancer proliferation and survival but renders cancer cells highly susceptible to perturbations within the metabolic network [5].

Lipid metabolism is frequently altered in human cancer [6, 7]. While most normal adult tissues use dietary lipids provided by the blood stream, many cancers show increased rates of de novo fatty acid (FA) biosynthesis [8]. The expression of most enzymes involved in FA biosynthesis is controlled by the sterol regulatory element binding proteins (SREBPs) and can be induced downstream of the PI3-kinase/Akt/mTORC1 signalling pathway in cancer [9]. Consequently, de novo FA biosynthesis is generally high in those tumour types that exhibit activating mutations within this pathway, including breast and prostate cancers [10, 11].

Several enzymes within the FA biosynthesis pathway have been found to be essential for cancer cell growth and survival and are currently pursued as targets for therapeutic development [6]. The potential selectivity of targeting lipid metabolism in cancer was also supported by a systems biology study employing a genome scale model of cancer metabolism that predicted specific dependence of cancer cells on lipid metabolism enzymes [12]. This suggests that targeting enzymes involved in these processes should selectively interfere with the growth of cancer cells without overt toxicity towards normal tissues.

Here, we show that cancer cells are highly dependent on stearoyl-CoA desaturase (SCD), the enzyme that introduces a double bond at the Δ9 position of newly synthesised FAs. Loss of cell number was only detected under lipid-depleted conditions and fully restored by exogenous mono-unsaturated FAs. Depletion of SCD caused specific alterations in lipid composition, indicating reduced availability of unsaturated acyl chains for the synthesis of phosphoglycerides and cardiolipins. Inhibition of SCD led to the release of cytochrome C from the mitochondria leading to the induction of apoptosis and increased sensitivity towards cytotoxic drugs and inhibitors of mitochondrial respiratory complexes. The sensitivity of cancer cells towards SCD inhibition was greatly increased by spheroid culture, a condition that recapitulates nutrient and oxygen gradients found in tumours. Expression of SCD is enhanced in human breast cancer tissue and associated with high disease grade. Finally, silencing of SCD resulted in efficient reduction of tumour growth in prostate orthografts. Together, these results indicate that SCD is an important node in the metabolism of cancer cells and that inhibition of this enzyme could provide a successful strategy for cancer treatment.

## Methods

### Antibodies and reagents

SCD inhibitors were purchased from Biovision and Caymen chemicals. Anti-SCD antibodies (SCD11-A) were from Alpha Diagnostics International and anti-Akt (9272), anti-cytochrome C (11940S) and anti-PARP (9542) from Cell Signalling. Hydrocortisone, EGF, Akt inhibitor (Akt V) and staurosporin were from Calbiochem. Insulin, cholera toxin, puromycin, doxycycline, paclitaxel and rotenone were from Sigma-Aldrich and metformin from Tocris Biosciences.

### Cell culture

RWPE1 and LNCaP (clone FGC) were obtained from the American Type Culture Collection. All other cell lines were obtained from LRI Cell Services (CRUK LRI, London, UK). All cell lines were authenticated using STR profiling and used at low passage. RWPE1 cells were grown in keratinocyte serum-free medium (Gibco) supplemented with epidermal growth factor and bovine pituitary extract (KGM). DU145, LNCaP and PC3 cells were grown in RPMI supplemented with 2 mM L-glutamine and penicillin/streptomycin. All breast cancer cell lines were grown in DMEM/F12 supplemented with serum, glutamine and penicillin/streptomycin. MCF10a cells were grown in DMEM/F12 supplemented with 5 % horse serum, 20 ng/ml EGF, 5 μg/ml hydrocortisone, 10 μg/ml insulin and 100 ng/ml cholera toxin.

### RNA interference

Breast and prostate cancer cells were reverse-transfected with 37.5 nM of Dharmacon siGENOME siRNA using Lullaby reagent (Oz biosciences). After 24 h, culture medium was replaced with either 10 % (full serum) or 1 % (low serum) foetal calf serum (FCS) containing medium. Additional supplementations were included for the experiments indicated. After 72 h, cells were fixed in 80 % ethanol over night at −20 °C. Plates were subsequently stained with DAPI (Sigma), and cell number was determined using the ACUMEN X3 microplate cytometer.

### Generation of doxycycline-inducible TetOnPLKO-shRNA cell lines

shRNA sequences targeting *SCD* or non-targeting control (NTC) were cloned into the TetOn-pLKO-puro lentiviral vector [13]. Clone IDs for shRNAs are as follows: shSCD #1 (TRCN0000056613) and shSCD #2 (TRCN0000056614). Lentiviruses were produced by cotransfecting HEK293T cells with lentiviral and packaging plasmids pCMVΔR8.91 and pMD.G. Supernatants were collected 72 h after transfection, mixed with polybrene (8 μg/mL) and used to infect cells. Cells were selected in medium containing puromycin (2 μg/mL).

### RNA extraction, reverse transcription, RT-qPCR

Total cell RNA was extracted using an RNeasy kit (QIAGEN); 2 μg of RNA was utilized for first strand cDNA synthesis with oligo-dT primers and Superscript II Reverse Transcriptase (Invitrogen). RT-qPCR was performed using SYBR® Green PCR Master Mix (Applied Biosystems) and Quantitect primers (QIAGEN) on an ABI Prism 7900 (Applied Biosystems). All reactions were performed in duplicate, and relative mRNA expression was calculated using the comparative Ct method after normalization to the loading control B2M.

### Protein analysis

Cells were lysed in Triton lysis buffer (1 % Triton X100, 50 mM Tris pH7.5, 300 mM NaCl, 1 mM EDTA, 1 mM DTT, 1 mM NaVO4, Protease-Inhibitor-Cocktail and Phosphatase-Inhibitor-Cocktail (Roche)). Proteins were separated on SDS-PAGE and blotted onto PVDF membrane (Immobilon). Membranes were blocked with 3 % bovine serum albumin (BSA) and incubated with antibody solutions, and signals were detected using ECL-reagent.

### Lipidomic analysis

Stable isotope labelling was performed as in [14]. For lipidomic analysis, lipids were extracted using a methanol/chloroform extraction method and quantified by LC-MS analysis on a Shimadzu IT-TOF LC/MS/MS system. Accurate mass (with mass accuracy ~5 ppm) and tandem MS were used for molecular species identification and quantification. The identity of lipids was further confirmed by reference to appropriate lipid standards. Cell pellets were spiked with appropriate internal standards (for each sample, 100 ng 12:0/12:0/12:0-TG, 200 ng 12:0/12:0-DG, 100 ng 12:0-MG, 200 ng 17:0-FA, 100 ng C17-Cer, 50 ng C17-SG, 200 ng 14:0/14:0/14:0/14:0-CL, 100 ng 12:0/12:0-PG, 200 ng 12:0/12:0-PE, 200 ng 12:0/12:0-PS, 400 ng 17:0/20:4-PI, 100 ng 12:0/12:0-PA, 400 ng 12:0/12:0-PC, 100 ng 17:0-LPA, 100 ng 17:0-LPC, 100 ng 12:0-Cer1P, 100 ng C17-S1P, 200 ng C17-SM and 50 ng C17-SPC) before extraction. The samples were extracted using a modified Folch method: first extraction with 4 ml chloroform:2 ml methanol:2 ml 0.88 % NaCl for each sample and second extraction of upper phase with 3 ml of synthetic lower phase of chloroform/methanol/0.88 % NaCl 2:1:1; the combined lower phases of the lipid extract were dried using a Thermo SpeedVac at room temperature under vacuum and re-dissolved in 50 μl chloroform/methanol 1:1, of which 7 μl was injected onto the column for LC-MS analysis. For LC/MS/MS analysis, a Shimadzu IT-TOF LC/MS/MS system hyphenated with a five-channel online degasser, four-pump, column oven, and autosampler with cooler Prominence HPLC (Shimadzu) was used. In detail, lipid classes were separated on a normal phase silica gel column (2.1 × 150mm, 4micro, MicoSolv Technology) using a hexane/dichloromethane/chloroform/methanol/acetanitrile/water/ethylamine solvent gradient based on the polarity of head group. Accurate mass (with mass accuracy ~5 ppm) and tandem MS were used for molecular species identification and quantification. The identity of lipid was further confirmed by reference to appropriate lipid standards. IT-TOF mass spectrometer operation conditions: ESI interface voltage +4.5 kv for positive ESI and −4 kv for negative ESI, heat block temperature 230 °C, nebulising gas flow 1.4 L/min, and CDL temperature 210 °C, with drying gas on at pressure of 100 kPa. All solvents used for lipid extraction and LC/MS/MS analysis were LC-MS grade from Fisher Scientific. Lipid amounts were normalised by protein concentrations of each sample.

### Crystal violet staining

Cells were seeded on 12-well plates. After incubation, cells were fixed with 70 % ethanol, stained with 0.01 % crystal violet. For quantification, dye was extracted with 10 % acetic acid and OD was measured at 560 nm.

### BrdU incorporation and apoptosis assays

Cells were labelled with BrdU for 1 h and analysed by fluorescence-activated cell sorting (FACS). For detection of apoptosis, cells were detached with trypsin and stained with Annexin V-pacific blue and propidium iodide (PrI). Relative proportion of viable cells and cells in early or late apoptosis were determined by FACS analysis.

### Oxygen consumption rates

Experiments were performed in a 96-well format using a SeahorseBioscience XF96 Extracellular Flux Analyser (Software Version 1.4) in assay medium supplemented with 1 mM sodium pyruvate and 10 mM glucose, with pH adjusted to 7.4. During the experiment, 1.264 μM oligomycin A (Sigma), 0.4 μM FCCP (Sigma) and 1 μM rotenone (Sigma) were injected. Oxygen consumption rates (OCR) were normalised to cell number.

### Spheroid growth assays

Cells were mixed with 2 % matrigel (BD Biosciences 356231) in culture medium and placed in 96-well ultralow attachment plates (Costar). Spheroid formation was initiated by centrifugation at 850×*g* for 10 min. Fresh growth medium was administered every 72 h. Spheroid size was determined by automated imaging on an inverted microscope (Axiovert 100 M, Carl Zeiss). Spheroids were fixed in neutral buffered formalin (NBF), suspended in 2 % agarose and paraffin embedded. Sections were incubated twice for 3 min in xylene, twice for 3 min in 100 % ethanol, twice for 2 min in 95 % industrial methylated spirit (IMS), twice for 2 min in 70 % IMS and rehydrated for 2 min in 50 % IMS before immunohistochemical analysis.

### Immunohistochemistry

Tissue microarray (TMA) slides (BR1921a) were purchased from US Biomax, Inc. (Rockville, MD, USA). Antibody staining was performed as in [14]. Legal consent was obtained by the company prior to collection of material.

### Comparative expression and survival analysis

Comparative expression analysis was performed using Oncomine (Compendia Bioscience, Thermo Fisher Scientific, Grand Island, NY, USA). Correlations between *SCD* expression and relapse-free survival in breast cancer were calculated using the GOBO analysis tool [15].

### Prostate orthograft tumour model

All the animal experiments conducted for this study were carried out with ethical approval from University of Glasgow under the revised Animal (Scientific Procedures) Act 1986 and the EU Directive 2010/63/EU (PPL 30/3185). Animals were housed in individual ventilated cages in a barrier facility proactive in environmental enrichment. Balb/C-nude male mice were obtained from Charles River Research Models and Services (UK). A midline lower abdominal incision was made on mice anesthetized by isoflurane inhalation. Using a 1-cc syringe with a 27-gauge needle, 2 × 10^6^ cells in 50 μl of serum-free phenol red-free glutamine containing RPMI were injected in one of the anterior prostate lobes. Mice were gavaged daily (0.2 ml) with doxycycline (10 mg/ml) starting at 10 days post-surgery or as indicated. Mice were anaesthetised, intraperitoneally injected with VivoGlo™ Luciferin (Promega) and imaged using an IVIS Spectrum imaging system. Images were analysed using the IVIS Living Image software. Ultrasound imaging was carried out in three-dimensional (3D)-mode using Vevo® 3100 Imaging System (Visual Sonics).

## Results

### Targeted RNAi screen identifies SCD as an essential gene in breast and prostate cancer cells

**Fig. 1:**
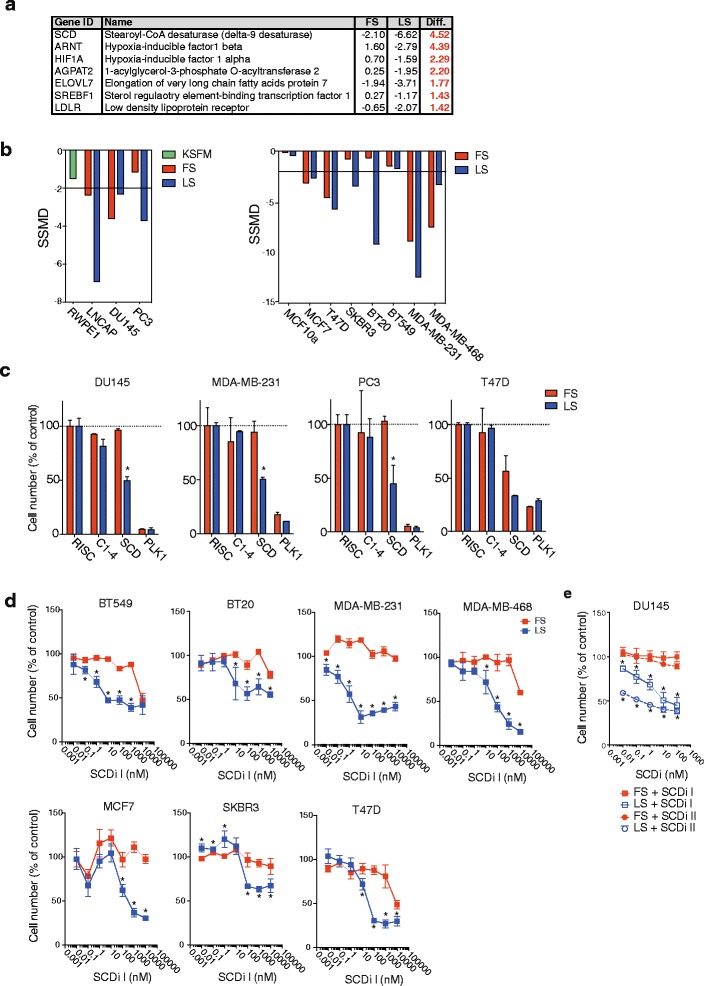
SCD ablation is detrimental to breast and prostate cancer cell lines cultured in low serum. **a** Strictly standardised mean difference (SSMD) calculated from cell numbers obtained in a functional genomic screen targeting lipid metabolism genes. Genes with the strongest differential (Diff.) between full serum (10 % FCS, FS) and low serum (1 % FCS, LS) are shown. **b** Screen results for SCD RNAi across the panel of breast and prostate cell lines. SSMD values below −2 indicate statistical significance. RWPE1 cells were cultured in KSFM. **c** Loss of cell number following silencing of SCD or PLK1 in DU145, MDA-MB-468, PC3 and T47D cells cultured in full serum (FS) or low serum (LS) for 72 h. Cell numbers are relative to non-targeting controls (RISC). Statistical comparisons were performed using Student *t* test (*p ≤ 0.05). **d** Breast and prostate cancer cell lines were treated with different doses of SCD inhibitor for 72 h in medium containing full (FS) or low (LS) serum. Cell numbers are relative to untreated controls. Data represent the mean ± SEM of three independent biological replicates. Statistical comparisons were performed using Student *t* test (*p ≤ 0.05). **e** DU145 cells were treated with increasing concentrations of two structurally distinct SCD inhibitors (SCDi I and SCDi II) for 72 h in medium containing full (FS) or low (LS) serum. Cell number is relative to untreated controls. Data represent the mean ± SEM of three independent biological replicates. Statistical comparisons were performed using Student *t* test (*p ≤ 0.05)

To identify genes selectively important for biomass accumulation in cancer cells, while not impacting on energy production in non-transformed cells, we employed an unbiased screen of 66 genes involved in lipid metabolism. These genes have been previously predicted to be essential for cancer cells [12]. We have previously reported a functional genomic screen in seven breast and three prostate cancer cell lines, representative of different disease subtypes (Additional file 1: Figure S1), and two non-transformed cell lines from the same tissue (MCF10a and RWPE1, respectively), which determined the effect of gene silencing on the total cell number [14]. To expose the selective vulnerabilities of cancer cells likely to be maintained in vivo [16–18], conditions of reduced availability of serum-derived factors were included. Comparison of the screen results obtained in full and low serum revealed differential effects after silencing of several genes, with the strongest difference observed for SCD (Fig. 1a). Depletion of SCD in low serum caused a significant reduction in cell number in nine out of the ten cancer cell lines tested, while having little or no effect on the two non-cancer cell lines, RWPE1 and MCF10A (Fig. 1b). These findings were confirmed using independent siRNA sequences in four representative cancer cell lines. Depletion of SCD resulted in a substantial reduction in cell number when cells were grown in low serum (Fig. 1c). In contrast, depletion of SCD in full serum only reduced cell numbers in T47D cells while having little or no effect on the other three lines (Fig. 1c). Low serum conditions did not influence the cytotoxicity of depleting the G2/M cell cycle transition protein polo-like kinase 1 (PLK1) (Fig. 1c).

To confirm the reduction in cell number observed after SCD silencing is due to loss of enzyme activity, we used two structurally unrelated SCD inhibitors: 4-(2-chlorophenoxy)-*N*-(3-(3-methylcarbamoyl)phenyl)piperidine-1-carboxamide (SCDi I) and 3-[4-(2-chloro-5-fluorophenoxy)-1-piperidinyl]-6-(5-methyl-1,3,4-oxadiazol-2-yl)-pyridazine (SCDi II). Treatment with SCDi I reduced proliferation of breast and prostate cancer cell lines grown in low serum in a dose-dependent manner, while having almost no effect in full serum (Fig. 1d), thus phenocopying the effect of gene silencing. Similar results were obtained with SCDi II in DU145 cells (Fig. 1e). Moreover, silencing of SCD using two different shRNA hairpins also efficiently reduced cell numbers of DU145 cells grown in low serum (Additional file 1: Figure S1b and c). Together, these results implicate SCD in growth and survival of cancer cells under serum-deprived conditions.

### SCD expression is high in human prostate and breast cancers and correlates with tumour grade

**Fig. 2:**
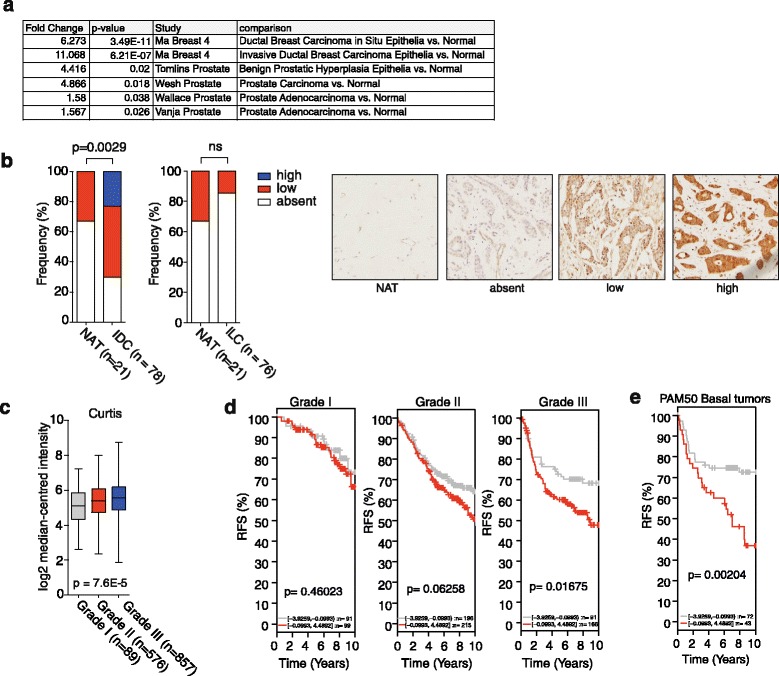
SCD expression is increased in breast and prostate cancers and associated with poor survival. **a** Differential expression of SCD between breast and prostate cancers and normal tissue found in different public datasets (Oncomine). **b** Immunohistochemical analysis of SCD in breast cancer and normal adjacent tissue (NAT) samples from invasive ductal carcinoma (IDC) and invasive lobular carcinoma (ILC). SCD staining intensity was classified as absent, low or high. Representative images of NAT and IDC samples indicating staining intensities. **c** Correlation of SCD expression in IDC patient samples with tumour grade (Curtis dataset, Oncomine). **d** Kaplan Meier plots for relapse-free survival (RFS) stratified by SCD expression in breast cancers of different grades. **e** Kaplan Meier plots for RFS stratified by SCD expression in patients with basal-like breast cancers as determined by expression of the PAM50 signature

To investigate SCD expression in cancer, we queried published datasets of human cancer. We found that SCD was upregulated in ductal breast carcinoma in situ and invasive ductal breast carcinoma (IDC—the most common type of breast cancer) samples, compared to normal tissue (Fig. 2a). Benign prostate hyperplasia, prostate carcinoma and prostate adenocarcinoma samples also showed increased SCD expression (Fig. 2a). To confirm the expression of SCD protein in breast cancer, we performed immunohistochemical staining of a breast cancer tissue microarray containing a total of 192 cores representing different breast cancer subtypes. High SCD expression was detected in approximately 20 % of IDCs, while it was only detected at low levels in normal adjacent breast tissue (*p* = 0.0029) (Fig. 2b). In contrast, only weak SCD staining was detected in invasive lobular carcinomas (ILCs), the second most common breast cancer subtype (Fig. 2b). Increased expression of SCD in IDCs compared to other breast cancer subtypes was also confirmed in a published mRNA dataset representing the six most common breast cancer subtypes (Additional file 1: Figure S1d). We also found that expression of SCD increased with tumour grade (Fig. 2c and Additional file 1: Figure S1e) and was higher in patients with low overall survival (Additional file 1: Figure S1f). Finally, we found that high SCD expression is predictive of poor relapse-free survival in breast cancer. This was particularly pronounced in grade III tumours (Fig. 2d) and those expressing the PAM50 signature [19], indicative of the basal subtype (Fig. 2f). Basal breast cancers are predominantly of a high histological grade on detection (grade III) and have an increased probability of metastatic progression and formation of distal metastases [20]. Taken together, these results demonstrate that SCD is expressed in breast and prostate cancers and that high levels of SCD expression are indicative of advanced disease.

### Low serum conditions alter the cellular lipid profile of cancer cells

**Fig. 3:**
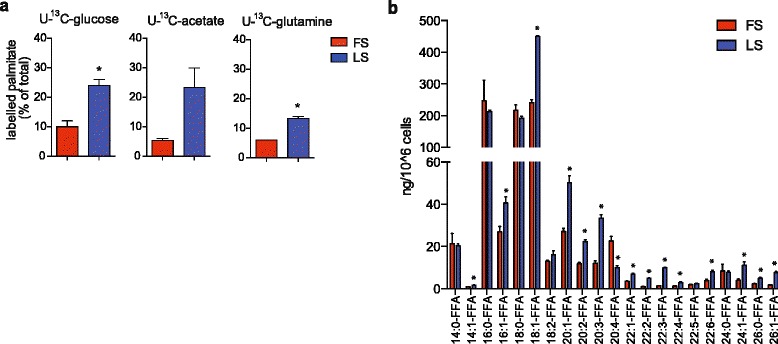
Low serum conditions induce de novo lipogenesis and alter the composition of cellular free fatty acids. **a** Incorporation of ^13^C-carbon from glucose, glutamine or acetate in full (FS) or low (LS) serum in DU145 cells. **b** Quantitative lipid profiling of FFA content of DU145 cells grown in full (FS) or low (LS) serum for 48 h using LC-MS/MS. Data represent the mean ± SEM of three biologically independent replicates. Statistical comparisons were performed using Student *t* test (*p ≤ 0.05)

To examine whether the effect of SCD depletion in low serum is a consequence of increased dependency on de novo lipogenesis, DU145 prostate cancer cells were cultured in media supplemented with stable isotopes of one of the three main carbon sources for lipid biosynthesis, ^13^C-glucose, ^13^C-acetate or ^13^C-glutamine. Labelling of intracellular palmitate was quantified using LC-MS-based metabolomics. In full serum, labelled palmitate derived from these precursors accounted only for a minor fraction of the total pool, ranging from 5 to 10 % (Fig. 3a). Culture in low serum conditions increased the incorporation from all precursors (Fig. 3a), indicating that serum depletion enhances de novo FA synthesis.

We next examined the composition of intracellular-free FAs (FFAs) in DU145 cells grown under full and low serum conditions. Strikingly, cells displayed high amounts and relative abundance of mono-unsaturated FFAs (Fig. 3b and Additional file 2: Figure S2a). In particular, the ratio between oleic acid (18:1) and stearic acid (18:0) was significantly higher in the intracellular FFA pool compared to serum FFAs (Additional file 2: Figure S2b), suggesting that mono-unsaturated FFAs are not derived from the serum FFA pool. However, it is also possible that a proportion of intracellular mono-unsaturated FFAs are derived from desaturated phospholipids, such as lysophosphatidylcholine, as demonstrated previously [21]. Interestingly, the levels of mono-unsaturated FFAs were further increased when cells were cultured in low serum (Fig. 3b), suggesting that the high abundance of unsaturated intracellular FFAs in cancer cells is a result of intrinsic desaturase activity and is increased under conditions when extracellular lipid supply is reduced and de novo FA synthesis is activated.

### SCD maintains fatty acid desaturation and viability in cancer cells

**Fig. 4:**
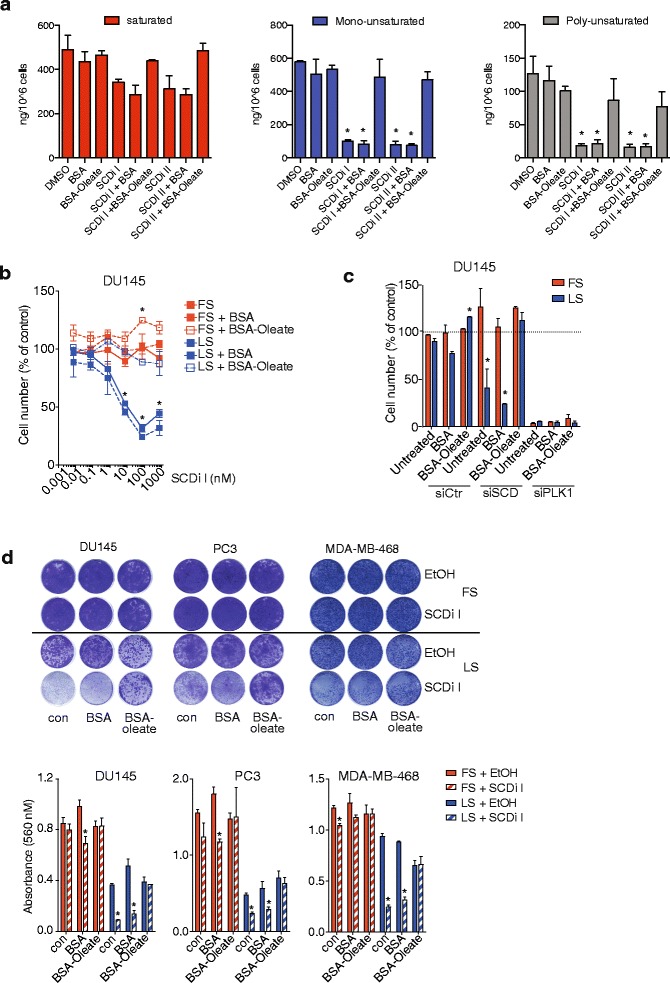
SCD maintains fatty acid desaturation to support viability of cancer cells. **a** DU145 cells were treated with SCD inhibitors (100 nM) or solvent (DMSO) for 48 h in medium with low serum or supplemented with BSA or BSA-coupled oleic acid (BSA-oleate). Quantitative lipid profiling of free fatty acids (FFA) content was performed by LC-MS/MS. Concentrations of saturated, mono-unsaturated and poly-unsaturated FFA are displayed. Data represent the mean ± SEM of three biologically independent replicates. Statistical comparisons were performed using Student *t* test (*p ≤ 0.05). **b** DU145 cells were treated with increasing concentrations of SCD inhibitor for 72 h in medium containing full (FS) or low serum (LS) or medium supplemented with BSA or BSA-oleate. Cell numbers are normalised to solvent treated controls. Statistical comparisons were performed using Student *t* test (*p ≤ 0.05). **c** DU145 cells were transfected with siRNA targeting SCD (siSCD), PLK1 (siPLK1) or non-targeting controls (siCtrl). Cells were cultured for 72 h in medium containing full (FS) or low serum (LS) or medium supplemented with BSA or BSA-oleate. Cell numbers are displayed relative to untreated siCtrl transfected cells. Data represent the mean ± SEM of two biologically independent experiments with two replicates each. Statistical comparisons were performed using Student *t* test (*p ≤ 0.05). **d** DU145, PC3 and MDA-MB-468 cells were treated with SCD inhibitor (SCDi I) or solvent (DMSO) under full (FS) or low (LS) serum conditions for 10 days. Cells were stained using crystal violet and quantified. Data represent mean ± SEM of two biologically independent experiments. Statistical comparisons were performed using Student *t* test (*p ≤ 0.05)

To establish the consequences of SCD inhibition on cellular lipid composition, we determined concentrations of saturated, mono-unsaturated and poly-unsaturated FFAs in DU145 prostate cancer cells following SCD inhibition in low serum conditions (Additional file 3: Figure S3a). As expected, levels of mono- and poly-unsaturated FFA species were significantly reduced, while levels of saturated FFAs were less affected (Fig. 4a and Additional file 3: Figure S3b). Consequently, this caused an increase in the relative proportion of saturated FFA in the total FFA pool (Additional file 3: Figure S3c and d).

We next investigated the effect of restoring levels of mono-unsaturated FFA on cellular lipid pools by providing cells with exogenous oleic acid at the same concentration found in full medium. Efficient coupling of oleic acid to BSA was confirmed by mass spectrometry (Additional file 2: Figure S2c). Interestingly, addition of oleic acid fully restored the amounts of mono- and poly-unsaturated FFAs in the cells (Fig. 4a and Additional file 3: Figure S3b) and reduced the relative proportion of saturated FFAs in the cellular FFA pool (Additional file 3: Figure S3d).

We also analysed acyl-chain length and composition of membrane phosphoglycerides in cancer cells cultured in full or low serum. Lipid species containing mono-unsaturated acyl-chains showed a marked increase in the amount in cells cultured in low serum (Additional file 4: Figure S4), suggesting that newly synthesised FAs contribute to the phosphoglyceride pool under those conditions. Inhibition of SCD caused a marked reduction in phosphatidylethanolamine (PE) containing mono-unsaturated acyl-chains, with the major two species, 36:1-PE and 36:2-PE, showing more than 50 % reductions (Additional file 5: Figure S5a and b). This resulted in a relative increase of long-chain poly-unsaturated PE species to the total pool (Additional file 5: Figure S5c). Interestingly, re-addition of oleic acid restored levels of mono-unsaturated PE (Additional file 5: Figure S5b), while reducing the relative proportion of some poly-unsaturated species (Additional file 5: Figure S5c). Similar results were also obtained for phosphatidylinositol (PI) and phosphatidylserine (PS) (Additional file 6: Figure S6 and Additional file 7: Figure S7, respectively).

To confirm that the loss in cell number after treatment with siRNA and SCDi against SCD was indeed due to reduced availability of mono-unsaturated FAs, we reasoned that supplementing cancer cells with oleic acid, the enzymatic product of the SCD reaction, would rescue cell survival. Indeed, supplementation with BSA-oleate completely restored cell viability in the presence of SCD inhibitor (Fig. 4b) and prevented the reduction in cell number following siRNA-mediated SCD silencing (Fig. 4c). Importantly, addition of BSA-oleate did not affect the viability of cells after silencing of PLK1, confirming the specificity of the assay (Fig. 4c). Moreover, supplementation of oleic acid not only provided immediate relief from SCD inhibition but also supported long-term maintenance of viable cells in the presence of the SCD inhibitor in low serum conditions (Fig. 4d). This suggests that maintaining a pool of mono-unsaturated FAs is essential for sustained cancer cell viability.

### Inhibition of SCD alters cardiolipin composition and sensitises cancer cells towards apoptosis

**Fig. 5:**
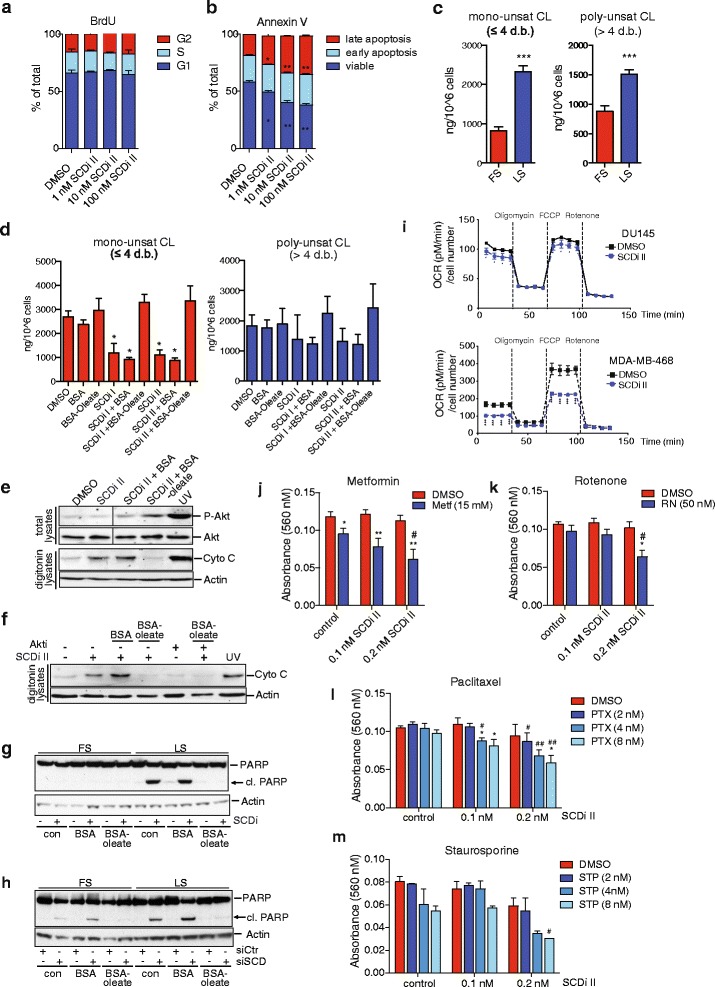
SCD inhibition alters cellular cardiolipin composition leading to cytochrome C release and sensitisation towards apoptosis. **a** DU145 cells were treated with different concentrations of SCD inhibitor (SCDi II) and proliferation was determined by BrdU labelling. **b** Cells treated as in **a** were used to determine apoptosis using Annexin V staining. Data represent mean ± SEM of three independent biological replicates. Statistical comparisons were performed using Student *t* test (*p ≤ 0.05). **c** Quantitative lipid profiling of cardiolipin (CL) content of DU145 cells grown for 48 h in medium containing full (FS) or low (LS) serum using LC-MS/MS. Concentrations of mono-unsaturated (≤4 double bonds) and poly-unsaturated (>4 double bonds) CL species are displayed. Data represent the mean ± SEM of three independent biological replicates. Statistical comparisons were performed using Student *t* test (***p ≤ 0.001). **d** DU145 cells were treated with SCD inhibitors (100 nM) or solvent (DMSO) for 48 h in medium containing low serum or medium supplemented with BSA or BSA-coupled oleic acid (BSA-Oleate). Quantitative lipid profiling of CL content was determined using LC-MS/MS. Concentrations of mono- and poly-unsaturated CL species are displayed. Data represent the mean ± SEM of three independent biological replicates. Statistical comparisons were performed using Student *t* tests (*p ≤ 0.05). **e** DU145 cells were treated with SCD inhibitor (SCDi I) or solvent (DMSO) for 48 h in medium containing low serum or medium supplemented with BSA or BSA-oleate. Cells were lysed by digitonin, and the presence of cytoplasmic cytochrome C was determined. UV treatment was used as positive control. Vinculin is shown as loading control. Levels of phosphorylated Akt (S473) and total Akt were detected in total lysates. **f** DU145 cells were treated as in **e** but 3 μM of Akt inhibitor was added prior to addition of BSA-oleate. Cytoplasmic cytochrome C was detected in digitonin lysates. **g** DU145 cells were treated with SCD inhibitor (SCDi I) or solvent (DMSO) in medium containing full (FS) or low (LS) serum or medium supplemented with BSA or BSA-oleate. The presence of full-length and cleaved (cl.) PARP was determined. Actin is shown as loading control. **h** DU145 cells were transfected with siRNA targeting SCD (siSCD) or non-targeting controls (siCtrl) and cultured as in **e**. The presence of full-length and cleaved (cl.) PARP was determined. Actin is shown as loading control. **i** DU145 or MDA-MB-468 cells were treated with SCD inhibitor (100 nM) for 48 h, and oxygen consumption rate (OCR) before and after addition of oligomycin, FCCP and rotenone was determined using a Seahorse Bioanalyzer. **j**–**m** DU145 cells were treated with the indicated doses of SCD inhibitor (SCDi II) either alone or in combination with different doses of metformin (Metf, **j**), rotenone (RN, **k**), paclitaxel (PTX, **l**) or staurosporin (STP, **m**) for 72 h in medium containing low serum. Cell viability was determined by crystal violet staining. Data represent the mean ± SEM of three independent biological replicates. Statistical comparisons were performed using Student *t* tests (*p ≤ 0.05, **p ≤ 0.01 compared to SCDi alone; #*p* ≤ 0.05, ##*p* ≤ 0.01 compared to no SCDi)

We next asked whether the reduction in cell number observed after SCD inhibition is due to reduced proliferation or induction of cell death. BrdU labelling revealed no change in cell proliferation after SCD inhibition (Fig. 5a). In contrast, we observed an increase in Annexin V-positive cells indicative of enhanced apoptosis (Fig. 5b). High levels of saturated FAs have been shown to induce apoptosis in breast cancer cells due to changes in cardiolipin (CL) turnover [22]. CLs are a class of structurally unique phospholipids that are localised in the inner membrane of mitochondria and have essential roles in the function of this organelle. Acyl chains of CL molecules are constantly remodelled, and their length and degree of unsaturation have a large influence on the function of CL-containing membranes [23].

We therefore investigated whether modulation of FA availability from serum alters composition of CL molecules in cancer cells. The major CL species in DU145 prostate cancer cells grown in full serum contain mainly shorter acyl-chains with one or two double bonds each (Additional file 8: Figure S8a). Cells cultured in low serum contain significantly higher levels of mono- and poly-unsaturated CL species (Fig. 5c), most prominently 70:4-CL and 72:4-CL (Additional file 8: Figure S8a), indicating that reduced availability of exogenous lipids and increased de novo lipid synthesis also affects the CL pool. Moreover, inhibition of SCD caused a substantial decrease in the levels of mono-unsaturated CL species, while poly-unsaturated CLs, particularly those with longer acyl-chains, were not affected (Fig. 5d and Additional file 8: Figure S8b). Importantly, addition of oleic acid restored the levels of mono-unsaturated CL species (Fig. 5d).

As mentioned above, CLs are integral components of mitochondrial inner membranes and control the release of cytochrome C during apoptosis [24]. Indeed, reduced CL synthesis in neonatal cardiomyocytes exposed to palmitate has been shown to cause the release of cytochrome C leading to apoptosis [25], indicating that CL composition not only modulates the cellular response to apoptotic stimuli but can also initiate cell death. We therefore investigated the effect of SCD inhibition on cytochrome C release and apoptosis in DU145 cells. Treatment with SCD inhibitor caused the efficient release of cytochrome C into the cytoplasm in cells grown in low serum (Fig. 5e). This was completely blocked by supplementing the cells with exogenous oleic acid (Fig. 5e), confirming that the release of cytochrome C is caused by a lack of mono-unsaturated FAs. In contrast to some previous reports [26–28], SCD inhibition did not alter Akt phosphorylation, presumably because the cells were cultured under reduced serum conditions. Instead, we noticed that treatment with oleic acid induced a small increase in Akt phosphorylation (Fig. 5e). However, pre-treatment with an Akt inhibitor did not block the ability of oleic acid to prevent cytochrome C release (Fig. 5f), indicating that oleic acid supports cell survival by restoring cellular lipid composition rather than altering Akt signalling. Moreover, induction of PARP cleavage in response to SCD inhibition or silencing was also completely blocked by oleic acid (Fig. 5g, h).

As cytochrome C is an integral component of the electron transport chain (ETC.), we also analysed the effect of SCD inhibition on oxygen consumption in cancer cells. Treatment of DU145 or MDA-MB-468 cells with SCD inhibitor in low serum not only reduced the basal OCR but also lowered maximal respiration in the uncoupled state, confirming that the activity of the ETC. is impaired in these cells (Fig. 5i). Moreover, SCD inhibition sensitised cancer cells towards metformin and rotenone, two inhibitors of complex I of the respiratory chain (Fig. 5j, k). We also investigated whether attenuation of SCD activity also increases the sensitivity of cancer cells towards drugs that induce apoptosis via the mitochondrial death pathway. While treatment with low doses of SCD inhibitor alone had little effect on cell viability, we found that it enhanced the toxicity of paclitaxel and staurosporine (Fig. 5l, m). Taken together, these results suggest that SCD inhibition causes alterations in the composition of lipids that are crucial for the function of the inner mitochondrial membrane and that altered lipid desaturation can enhance the susceptibility of cancer cells towards chemotherapeutic drugs and metabolic inhibitors.

### Spheroid cultures exhibit tumour-like lipid composition and depend on SCD activity

**Fig. 6:**
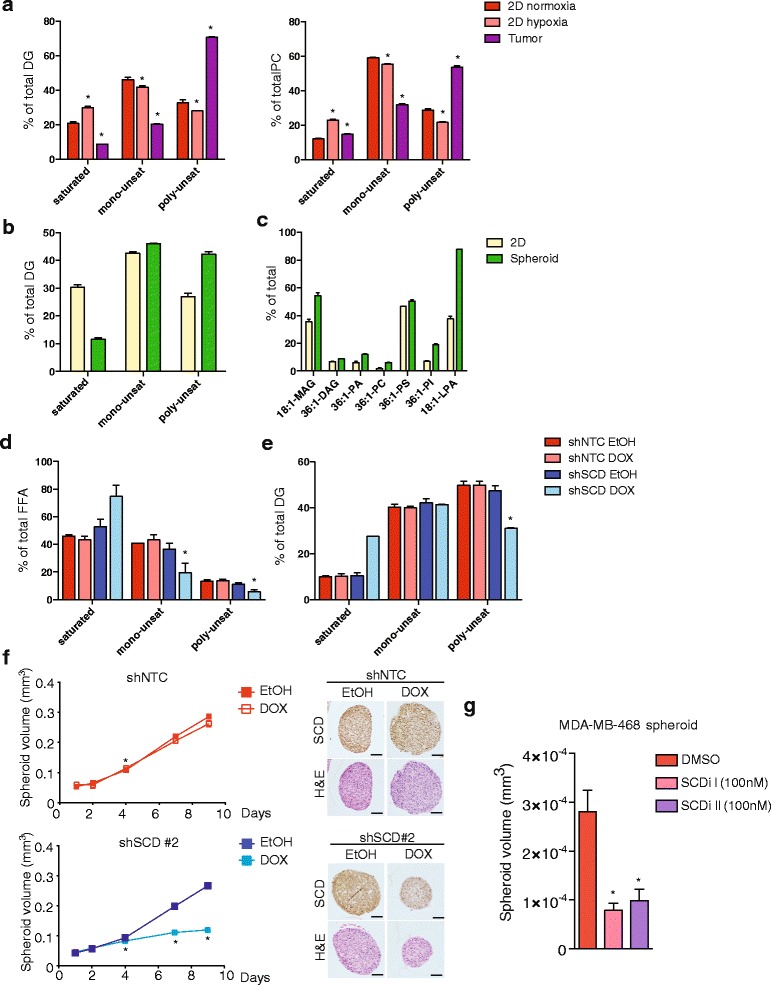
Spheroid culture reveals metabolic dependency of cancer cells on lipid desaturation. **a** BT474 cells were cultured in normoxia (20 % O_2_) or hypoxia (0.1 % O_2_) for 48 h before lipids were extracted and used to determine the relative amount of diacylglycerol (DG) and phosphatidylcholine (PC) species by LC-MS/MS. Archival tissue from six individual orthotopic xenograft tumours of BT474 cells [14] was also analysed. Statistical comparisons were performed using Student *t* tests (*p ≤ 0.05). **b** Lipids were extracted from T47D cells grown in adherent cultures (2D) or as tumour spheroids. Relative distribution of unsaturated and mono- or poly-unsaturated DAG species are shown. **c** Selected mono-unsaturated lipid species from T47D cells grown in 2D or as tumour spheroids. **d**, **e** DU145 cells expressing inducible shRNAs targeting SCD (shSCD #2) or scrambled control (shNTC) were grown as spheroids in the presence of solvent (EtOH) or 0.5 μg/ml doxycycline (DOX). Spheroids taken at day 9 were subjected to quantitative lipid profiling and relative amounts of free fatty acids (FFA, **d**) and diacylglycerol species (DAG, **e**) were determined. Data represent the mean ± SEM of three independent biological replicates. Statistical comparisons were performed using Student *t* tests (*p ≤ 0.05). **f** Spheroid size was determined at the indicated times. Spheroids taken at day 9 were fixed and subjected to immunohistochemical staining of SCD and H&E staining. **g** Spheroids of MDA-MB-468 cells were treated with 100 nM of SCD inhibitors for 4 days and spheroid size was determined. Statistical comparisons were performed using Student *t* test (*p ≤ 0.05)

Many biosynthetic processes are modulated by the tumour microenvironment, which determines the availability of nutrient and oxygen for metabolic reactions. As the SCD reaction requires molecular oxygen, it is likely that lipid desaturation is affected by tumour hypoxia. To explore the effect of different environmental conditions on the saturation levels of cellular lipid species, we used BT474, a highly tumourigenic HER2- and ER-positive breast cancer cell line, which readily forms xenograft tumours. These cells were cultured under normoxic (20 % O_2_) and hypoxic (0.1 % O_2_) conditions as two-dimensional (2D) cultures and subjected to lipid analysis. We mainly focused on diacylglycerol (DG) and phosphatidylcholine (PC), two lipid classes that show rapid turnover of their acyl chains [24] and should therefore reflect the availability of different FA species for membrane synthesis in these cells. Surprisingly, altering oxygen concentrations only led to a small increase in saturated lipids and only a marginal reduction in mono- and poly-unsaturated DG and PC species (Fig. 6a and Additional file 9: Figure S9a and b). This indicates that these culture conditions alone do not compromise the availability of unsaturated FAs for lipid synthesis. We next analysed lipids extracted from BT474 cells grown as orthotopic xenograft tumours. Compared to cells cultured in 2D, tumours showed a marked increase in the relative amounts of DG and PC species containing two or more double bonds (Fig. 6a and Additional file 9: Figure S9a and b), suggesting that the in vivo environment induces synthesis and/or uptake of unsaturated lipids.

Cancer cells can be grown as multi-layered 3D spheroids. These structures recreate the nutrient and oxygen gradients that cancer cells are exposed to within a growing tumour. We found that T47D cells grown as tumour spheroids show a similar increase in poly-unsaturated DG species as found in tumours (Fig. 6b and Additional file 9: Figure S9c). Moreover, increased levels of several mono-unsaturated lipid species, most prominently PI and lysophosphatidic acid (LPA), were also detected (Fig. 6c). To examine the effect of SCD ablation on lipid composition in these conditions, we used DU145 cells expressing inducible shRNA hairpins targeting SCD to generate spheroids. These were cultured in full medium, and gene silencing was activated with doxycycline. As can be seen from the data shown in Fig. 6d, depletion of SCD caused a marked decrease in the relative amount of mono- and poly-unsaturated FFA species (Fig. 6d and Additional file 10: Figure S10a). Furthermore, the poly-unsaturated DG population, which was selectively upregulated in tumours (see Fig. 6a), was reduced when SCD was ablated (Fig. 6e and Additional file 10: Figure S10b). This was accompanied by a substantial reduction in spheroid size despite the presence of serum in the culture medium (Fig. 6f). A similar reduction on spheroid size was observed in MDA-MB-468 breast cancer cells following inhibition of SCD activity (Fig. 6g). Interestingly, this activity was seen at a concentration of the drug that was ineffective at inhibiting cell growth in 2D cultures in full serum (see Fig. 1d), suggesting that spheroid culture sensitises cancer cells towards SCD inhibition. These data confirm that SCD activity is essential to maintain levels of mono-unsaturated FAs in cancer cells exposed to the compromised metabolic environment created by 3D culture and that these FAs are rate limiting for cell growth under these conditions.

### SCD is essential for orthotopic growth of prostate cancers

Having shown that SCD is important to control lipid provision under conditions that resemble the metabolic microenvironment of tumours, we next investigated whether SCD expression is also essential for tumour growth. We implanted DU145 prostate cancer cells expressing inducible shRNAs targeting SCD orthotopically into the prostates of immunocompromised mice (nu/nu). Induction of gene silencing was induced with doxycycline. In one cohort, treatment was started 10 days after implantation when tumours were already detectable by bioluminescence imaging (early). In order to investigate the effect of SCD inhibition on established tumours, a second cohort was treated with doxycycline starting at day 47 post-implantation (late). Tumour growth was monitored over time, and mice were culled upon reaching the humane endpoint.

**Fig. 7:**
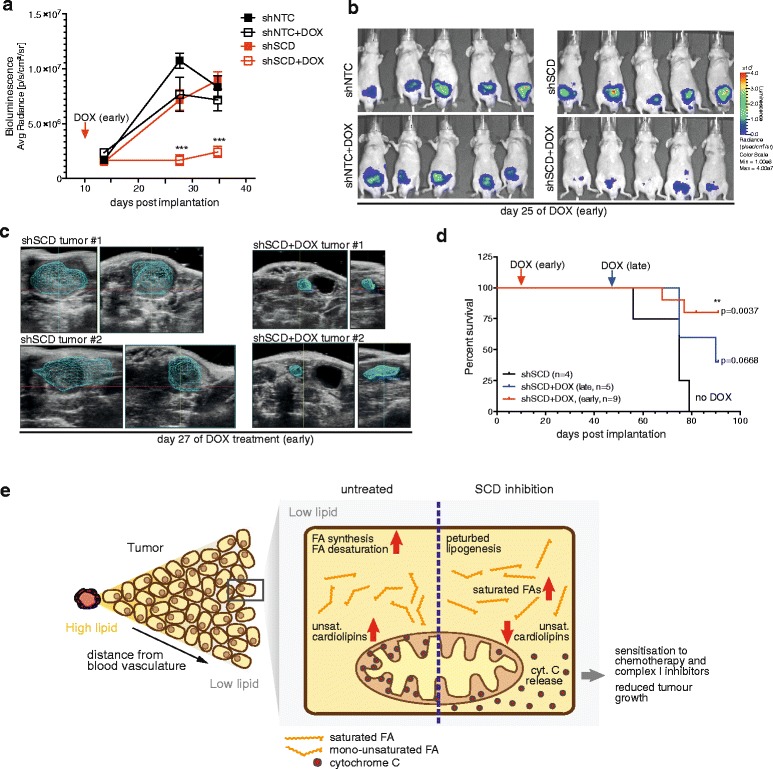
SCD is essential for orthotopic tumour growth of prostate cancer cells. **a** DU145 cells expressing inducible shRNAs targeting SCD (shSCD #2) or scrambled control (shNTC) were injected orthotopically into the frontal lobe of the prostate of immunocompromised mice (nu/nu). Gene silencing was induced 10 days post-implantation by treatment with doxycycline (DOX). Tumour growth was followed using intravital bioluminescence imaging of luciferase-positive cancer cells. Data represent mean bioluminescence ± SEM of eight mice per treatment group. Statistical comparisons were performed using Student t-tests (***p ≤ 0.0001). **b** Images of bioluminescence in mice at day 25 after initiation of doxycycline treatment. **c** Representative images of tumours detected by 3D ultrasound imaging 27 days after initiation of doxycycline treatment. **d** Survival curves of mice orthotopically implanted with prostate cancer cells and treated with doxycycline from day 10 onwards (early, *red line*) or day 47 onwards (late, *blue line*) compared to controls. Statistical comparisons were performed using the log-rank (Mantel-Cox) test (**p ≤ 0.001). **e** Schematic representation of the vulnerability of cancer cells towards inhibition of FA desaturation under the metabolically compromised conditions of the tumour microenvironment. Our data suggest that tumour cells are exposed to conditions of reduced availability of exogenous lipids, making them vulnerable towards inhibition of FA desaturation. Inhibition of SCD causes relative accumulation of saturated FAs and disturbs CL compositions resulting in release of cytochrome C, reduced mitochondrial activity, enhanced sensitivity towards chemotherapeutic drugs and reduced tumour growth

In the early treatment regimen, SCD ablation resulted in a substantial attenuation in tumour growth, while tumours in the untreated cohort, or the doxycycline-treated mice implanted with DU145 cells expressing non-targeting controls, continued to expand (Fig. 7a). Indeed, SCD silenced tumours were almost undetectable 25 days after doxycyline treatment was initiated (Fig. 7b). This difference in tumour size was confirmed by 3D sonographic imaging (Fig. 7c and Additional file 11: Movies S1–S8). Furthermore, SCD ablation also resulted in a significant increase in the life span of treated mice (Fig. 7d). In the late treatment group, SCD ablation did not have a major impact on tumour size as determined by bioluminescence imaging (data not shown). Nevertheless, SCD ablation still resulted in a discernable increase in survival compared to the untreated cohort (Fig. 7d). These results show that SCD is essential for the growth and survival of cancer cells exposed to the metabolic constraints of their native tissue environment.

In summary, our results demonstrate that SCD is an important enzyme to support the enhanced de novo lipid synthesis and desaturation in cancer cells under conditions where exogenous lipids are limiting. SCD controls the production of lipid species that are important for cell viability and determine the sensitivity of cancer cells towards chemotherapeutic agents. Finally, SCD is essential for tumour cell growth within the metabolic restraints of the tissue that they arise in.

## Discussion

Increased lipid biosynthesis is an important feature of cancer cells [6, 7, 29]. Inhibitors of fatty acid synthase (FASN) have so far shown limited therapeutic promise due to systemic toxicity [30]. Defining the exact contribution of lipid metabolism enzymes to cancer cell growth and survival is crucial to identify potential new therapeutic targets.

In this study, we analysed the response of breast and prostate cancer cell lines to the depletion of SCD, a rate-limiting enzyme for the production of mono-unsaturated fatty acids. We found that silencing of SCD reduced proliferation in almost all cancer cells lines studied without affecting the viability of non-malignant epithelial cell lines derived from the same tissues. In contrast to previous reports [31], we did not observe a cell cycle arrest in response to SCD inhibition. This could be because the cells were already cycling slower due to the reduced availability of serum-derived growth factors. Nevertheless, the strong and selective dependence of cancer cells on lipid desaturation observed here is in agreement with previous reports [31, 32]. It is possible that increased lipid desaturation maintains cellular functions that are selectively important in cancer cells. Indeed, lipid desaturation was shown to prevent engagement of the unfolded protein response in cancer cells due to their increased rates of protein synthesis [17, 18, 33].

SCD has been previously linked to cancer cell proliferation and survival [34, 35]. Increased expression of SCD has been found in several cancer types, including prostate [27, 36], liver [37], kidney [38] and breast cancer [39]. Inhibition of SCD by expression of antisense RNA reduced tumour formation in human lung adenocarcinoma cancer cells [26] while pharmacological inhibition of SCD was effective in blocking the growth of gastric and colon cancer cells [32, 36]. SCD also promotes proliferation and disease progression in prostate cancer by affecting cellular signaling cascades and modulating androgen receptor transactivation [27, 40]. Furthermore, it has been shown that breast cancers contain higher proportions of saturated and mono-unsaturated lipid species, indicative of increased lipid synthesis and desaturation rather than uptake of dietary lipids [41]. The same study also found reduced viability after SCD silencing in breast cancer cells [41], confirming the importance of this enzyme for cancer metabolism. Finally, altered serum lipids also offer diagnostic opportunities as reduced levels of triacylglycerides containing oleic acid were found to be indicative of prolonged survival in breast cancer patients after neoadjuvant treatment [42].

Our study provides additional evidence for the important role of SCD in cancer. We found that SCD is overexpressed in breast and prostate cancers compared to normal tissues. In breast cancer, high levels of SCD expression were confined to invasive ductal breast carcinomas but absent in invasive lobular carcinomas, providing important subtype specification. Moreover, SCD expression correlated with tumour grade and was indicative of disease relapse. SCD expression also determined reduced progression-free survival of high-grade tumours and those stratified by the PAM50 gene expression signature, which defines basal-like aggressive disease that presently has limited therapeutic options [19]. In keeping with this finding, three out of the four triple negative breast cancer cell lines used in this study showed high sensitivity towards SCD depletion.

Our study further demonstrates that the dependence of cancer cells on SCD is strongly determined by the availability of exogenous lipids. Lipid deprivation increased de novo lipid biosynthesis and enhanced the contribution of mono-unsaturated FAs to the cellular lipid pool. We also confirmed that the effect of SCD silencing on cancer cell viability was due to inhibition of enzymatic activity, as two structurally unrelated inhibitors of SCD activity showed comparable efficacy in reducing proliferation and survival in cancer cells. Both inhibitors reduced the levels of mono- and poly-unsaturated FA species and increased the relative abundance of saturated forms. This shift towards higher saturation could also be observed in the acyl chains of several membrane phosphoglycerides, indicating that SCD inhibition alters the composition of important cellular lipid species. However, the amounts of some phosphoglyceride species containing long-chain poly-unsaturated FAs were increased following SCD inhibition, suggesting that cells can remodel existing lipid pools or selectively take up certain lipid species when desaturation is blocked. Indeed, it has been shown that the selective uptake of unsaturated lyso-phospholipids is induced by hypoxia and in Ras-transformed cells [21].

Interestingly, we found that re-addition of oleic acid restored most of the alterations in lipid composition induced by SCD inhibition, confirming that this mono-unsaturated FA is indeed rate-limiting for the generation of a large part of the cellular lipid spectrum when exogenous lipids are scarce. Addition of oleic acid also prevented the loss of cell viability in response to SCD silencing or inhibition. This effect was maintained over several days, providing sufficient lipid substrate for membrane biosynthesis during this time. Of particular importance in this context is the continuous functionality of the inner mitochondrial membrane, which contains a high proportion of cardiolipins (CL). The degree of desaturation determines the function of this highly specialized lipid class, and the acyl chains in CL molecules undergo constant remodeling [23]. The composition of different CL molecules within the inner mitochondrial membrane can modulate the activity of ETC. complexes [24]. Moreover, the acyl chains of CL molecules interact with cytochrome c, thereby affecting both respiration and apoptosis [43].

We found that inhibition of SCD reduced levels of mono-unsaturated CL species, with a specific reduction in mono-unsaturated species. This was accompanied by the release of cytochrome C and induction of apoptosis. Sub-lethal doses of SCD inhibitor also increased the sensitivity of cancer cells towards chemotherapeutic agents and inhibitors of mitochondrial respiratory complexes. This is in agreement with a recent study showing that betulinic acid alters the saturation of CL species, causing mitochondrial damage and cytochrome C release, most likely through inhibition of SCD function [44]. It is also possible that the reported activation of AMP-activated protein kinase (AMPK) by SCD [45] could involve inhibition of mitochondrial activity by altering cellular CL composition.

We also observed that different culture conditions have substantial effects on lipid composition. Human cancer cells grown as orthotopic tumours exhibited specific lipid profiles that were characterised by high levels of mono- and poly-unsaturated lipid species. These lipid profiles were also detected in tumour spheroids, an experimental system that recreates the oxygen and nutrient gradients observed in tumors. Silencing of SCD reduced the ability of cancer cells to grow as spheroids, indicating that these conditions restrict the access to exogenous lipids making cancer cells dependent on SCD function. Cells within metabolically restricted hypoxic areas of human tumors frequently show therapy resistance and increased stem cell capabilities [46], making the selective targeting of this niche a therapeutic priority.

Most importantly, we found that depletion of SCD efficiently blocked the ability of prostate cancer cells to grow as orthotopic tumour xenografts, resulting in reduced tumour volume and prolonged survival of the host. This effect was also observed when SCD silencing was initiated after the tumours had already grown to a substantial size, indicating that targeting SCD could offer treatment opportunities in established cancer. It should be noted that the RNAi strategy used here selectively targets SCD only in cancer cells. This excludes potential effects on desaturase activity in cells of the tumour stroma or global alterations in the lipid metabolism of the host, which can contribute to tumour inhibition by systemic SCD inhibition.

## Conclusions

This study demonstrates that SCD is deregulated in human breast and prostate cancers and essential for cancer cell survival and tumour growth. Inhibition of SCD altered cellular lipid composition, leading to a distinct reduction in particular lipid species that depend on the availability of mono-unsaturated FAs. This phenotype was only observed under conditions when exogenous lipid sources were limited. The functional consequences of deregulated lipid metabolism in response to SCD inhibition included mitochondrial dysfunction, release of cytochrome C and induction of apoptosis, confirming the important role of fatty acid desaturation for essential cellular processes.

Taken together, our results highlight the importance of SCD for lipid provision in cancer cells under the metabolically compromised conditions that are likely to be encountered within the tumour microenvironment (Fig. 7e). We conclude that synthesis of mono-unsaturated FAs by SCD represents a metabolic bottleneck of lipid biosynthesis and thus provides a suitable target for therapeutic development.

## Additional files

Additional file 1: Figure S1. Cancer cell lines used in this study, effect of shRNA mediated silencing and expression of SCD in breast cancer. (PDF 876 kb) Additional file 2: Figure S2. Relative FFA content of DU145 cells grown in full or low serum, FFA content in FCS, BSA and BSA-oleate. (PDF 449 kb) Additional file 3: Figure S3. Spectrum of FFAs of cancer cells after SCD inhibition. (PDF 648 kb) Additional file 4: Figure S4. Comprehensive lipidomic analysis of cancer cells grown in full and low serum. (PDF 465 kb) Additional file 5: Figure S5. Analysis of phosphatidylethanolamine species in DU145 cells following SCD inhibition. (PDF 556 kb) Additional file 6: Figure S6. Analysis of phosphatidylinositol species in DU145 cells following SCD inhibition. (PDF 512 kb) Additional file 7: Figure S7. Analysis of phosphatidylserine species in DU145 cells following SCD inhibition. (PDF 496 kb) Additional file 8: Figure S8. Analysis of cardiolipin species in DU145 cells following SCD inhibition. (PDF 443 kb) Additional file 9: Figure S9. Lipid species in tumour spheroids resemble those in orthotopic breast xenografts. (PDF 533 kb) Additional file 10: Figure S10. Lipidomic analysis of DU145 tumour spheroids following SCD silencing. (PDF 497 kb) Additional file 11: Movies 1–8. Movies showing 3D reconstructions of ultrasound images of tumours after 27 days of doxycycline treatment. (ZIP 17982 kb)
