# Supplementary figures and images for: Inhibition of fatty acid desaturation is detrimental to cancer cell survival in metabolically compromised environments
Source: Cancer Metab. 2016 Apr 1;4:6. doi: 10.1186/s40170-016-0146-8 (PMC4818530; doi:10.1186/s40170-016-0146-8)

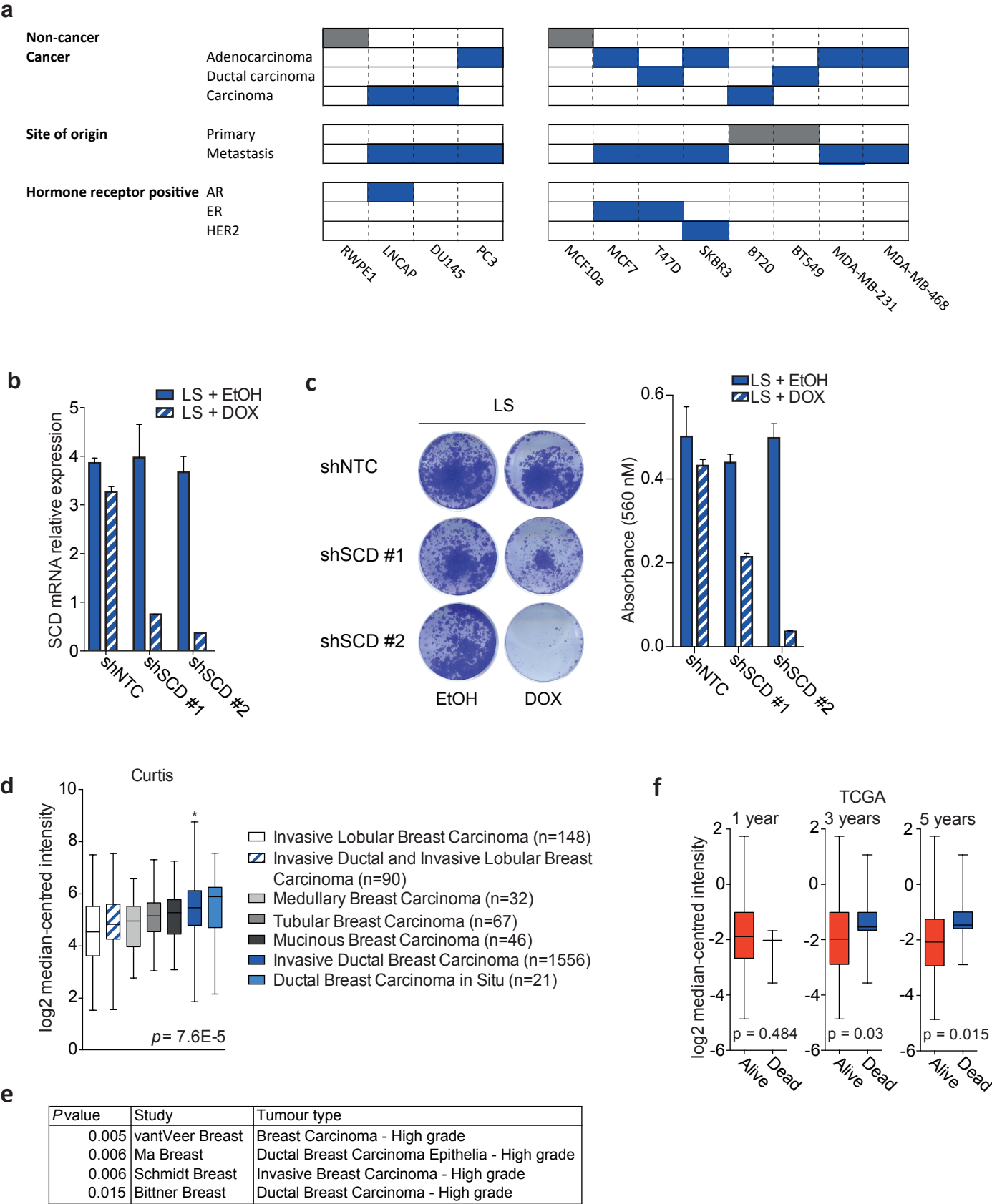

Supplement: Additional file 1: Figure S1. — Cancer cell lines used in this study, effect of shRNA mediated silencing and expression of SCD in breast cancer. (PDF 876 kb) [file 40170_2016_146_MOESM1_ESM.pdf]

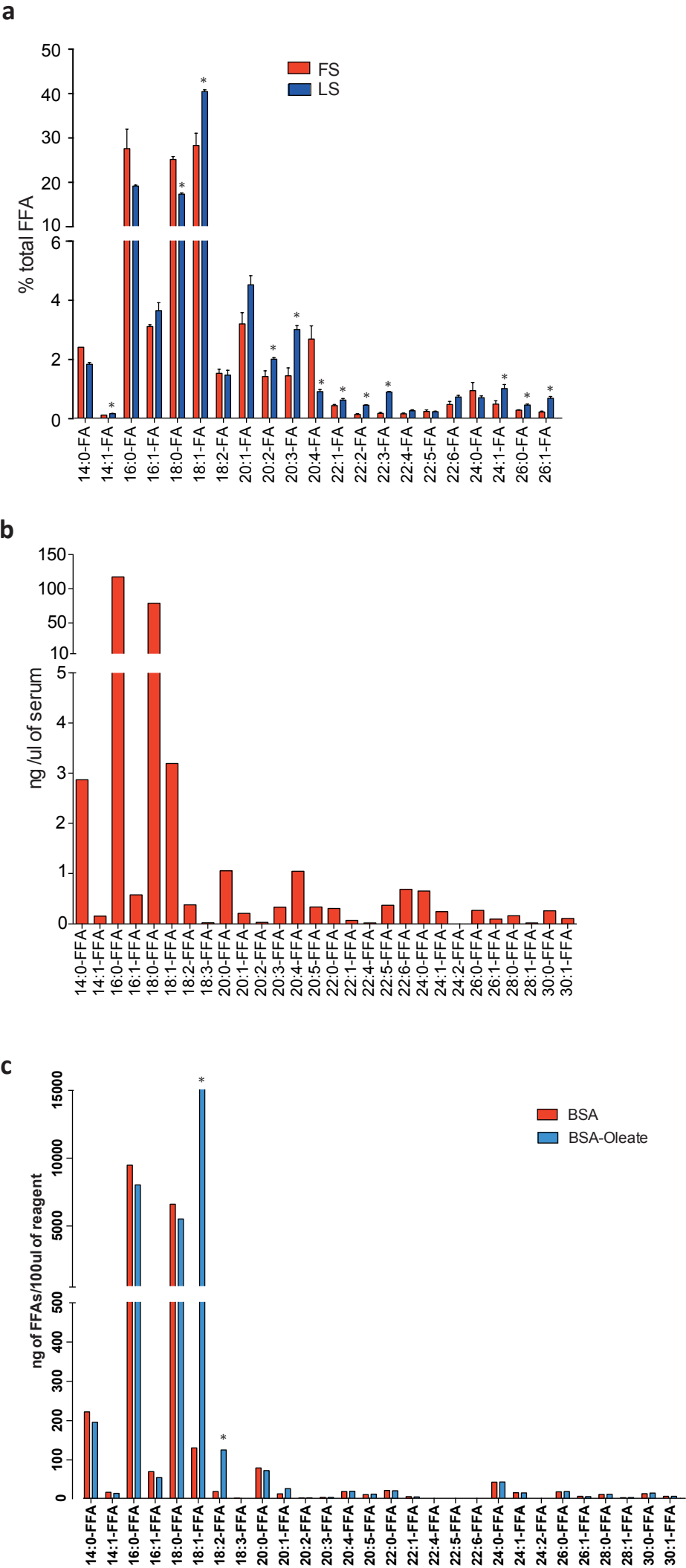

Supplement: Additional file 2: Figure S2. — Relative FFA content of DU145 cells grown in full or low serum, FFA content in FCS, BSA and BSA-oleate. (PDF 449 kb) [file 40170_2016_146_MOESM2_ESM.pdf]

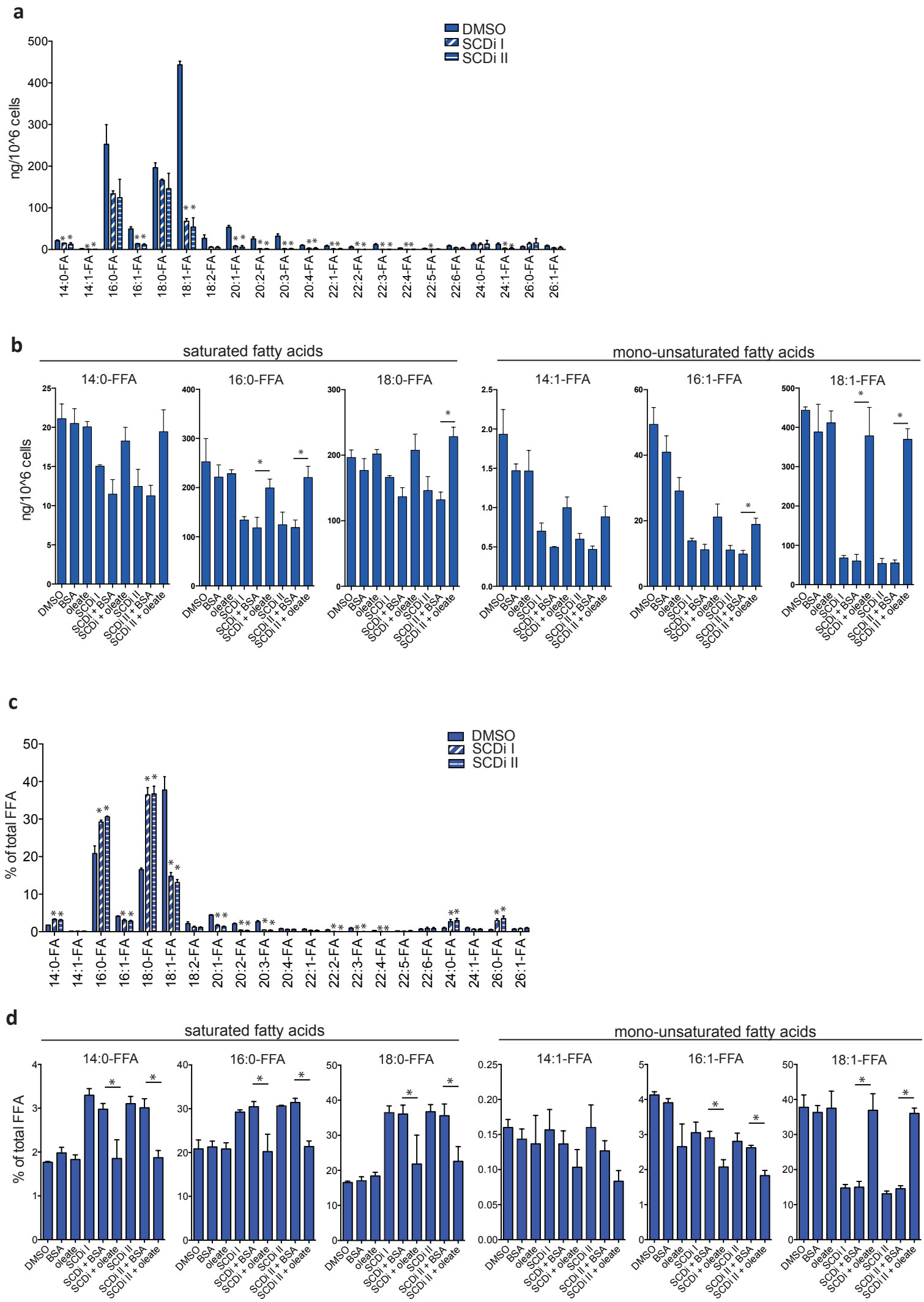

Supplement: Additional file 3: Figure S3. — Spectrum of FFAs of cancer cells after SCD inhibition. (PDF 648 kb) [file 40170_2016_146_MOESM3_ESM.pdf]

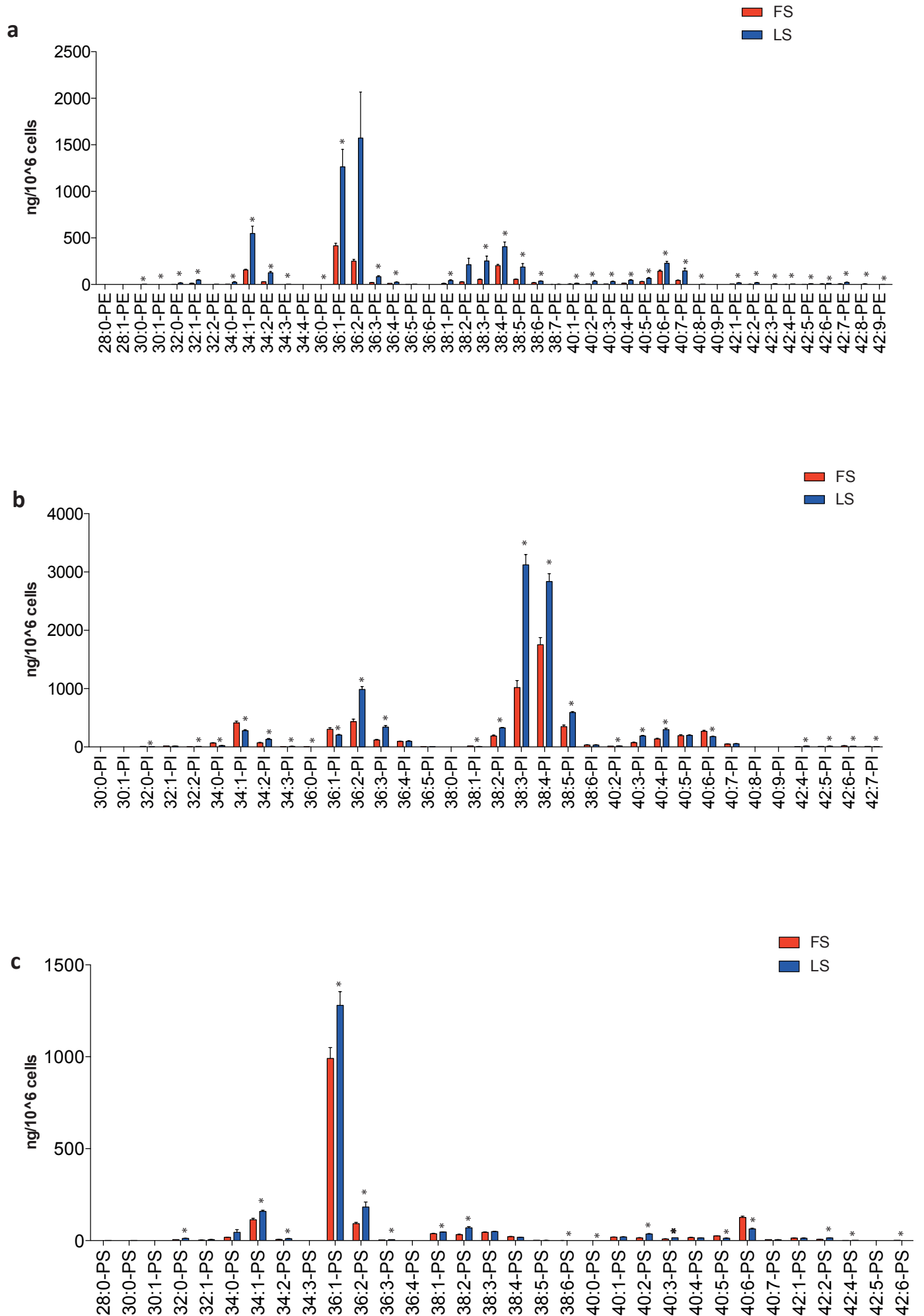

Supplement: Additional file 4: Figure S4. — Comprehensive lipidomic analysis of cancer cells grown in full and low serum. (PDF 465 kb) [file 40170_2016_146_MOESM4_ESM.pdf]

a

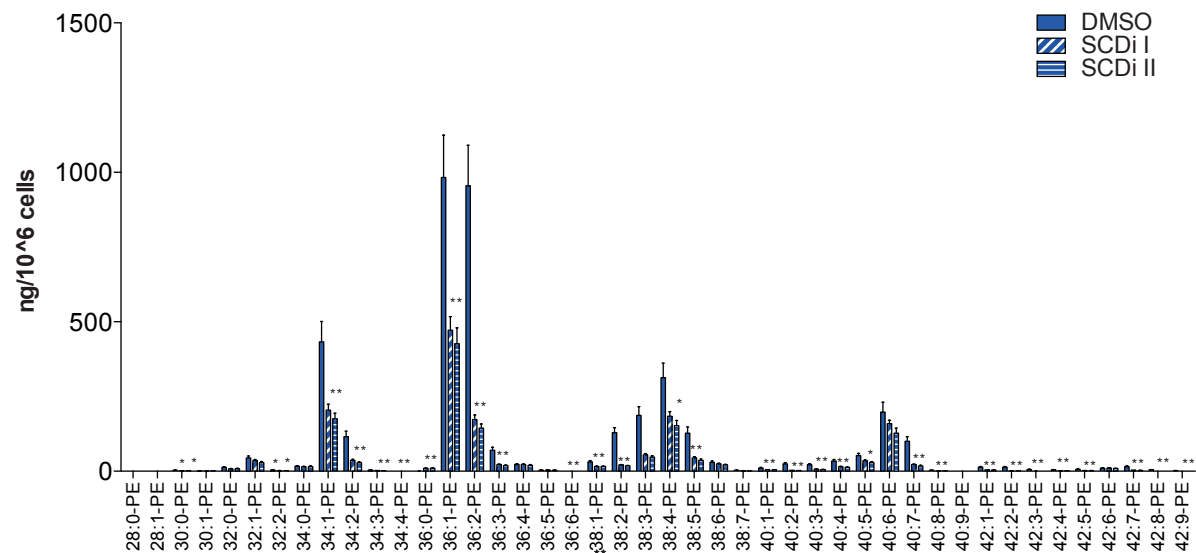

b

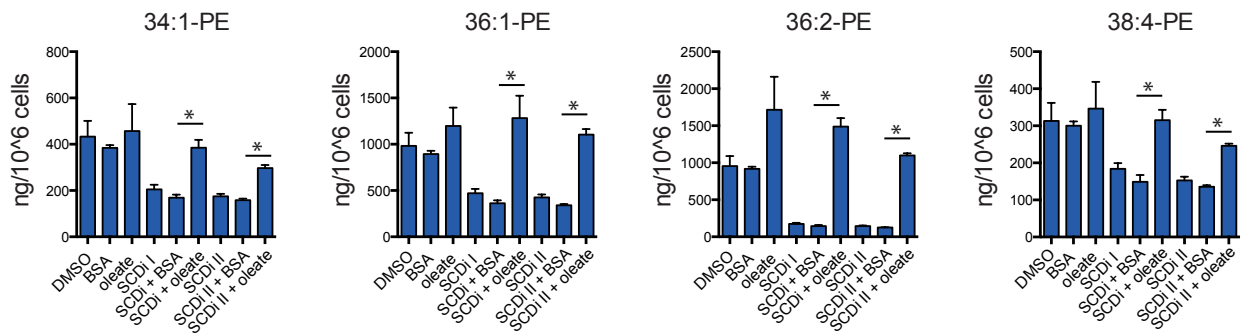

c

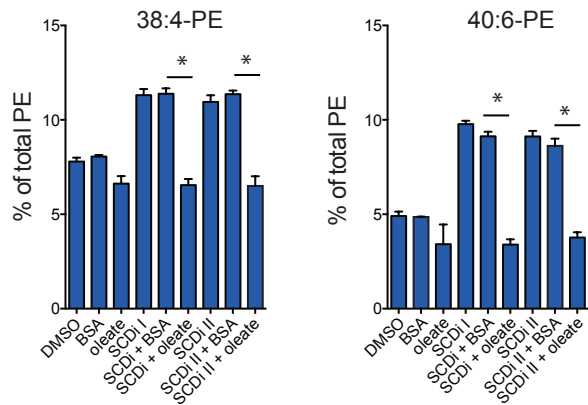

Supplement: Additional file 5: Figure S5. — Analysis of phosphatidylethanolamine species in DU145 cells following SCD inhibition. (PDF 556 kb) [file 40170_2016_146_MOESM5_ESM.pdf]

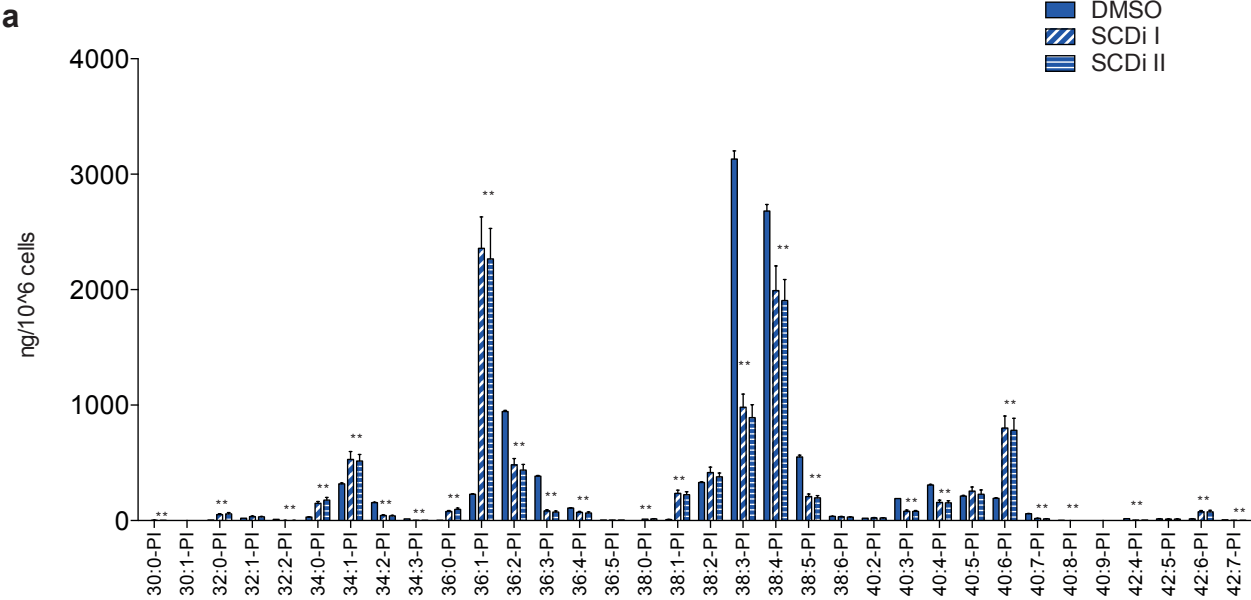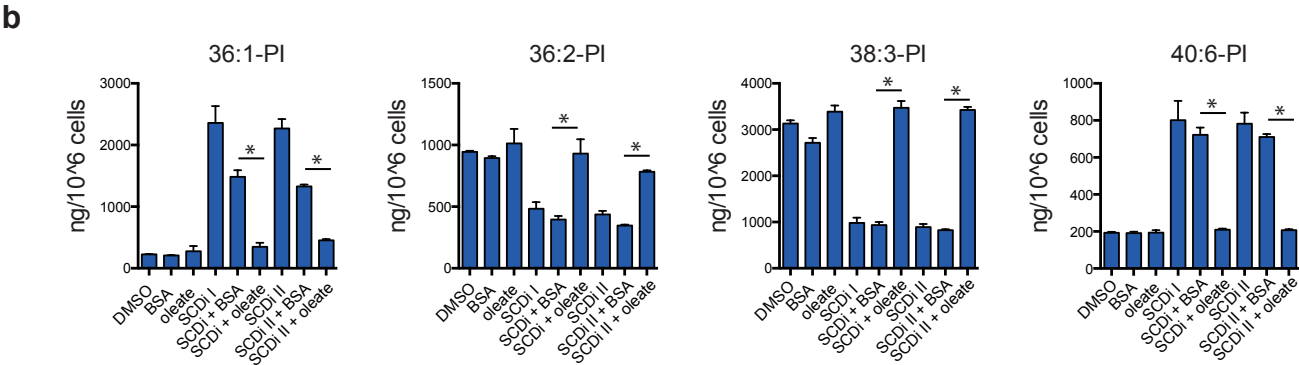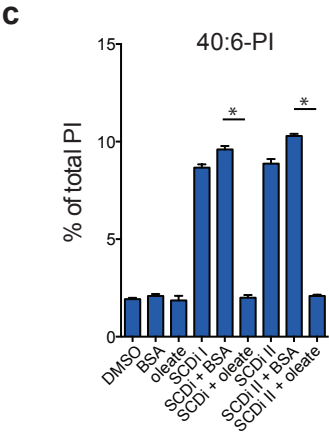

Supplement: Additional file 6: Figure S6. — Analysis of phosphatidylinositol species in DU145 cells following SCD inhibition. (PDF 512 kb) [file 40170_2016_146_MOESM6_ESM.pdf]

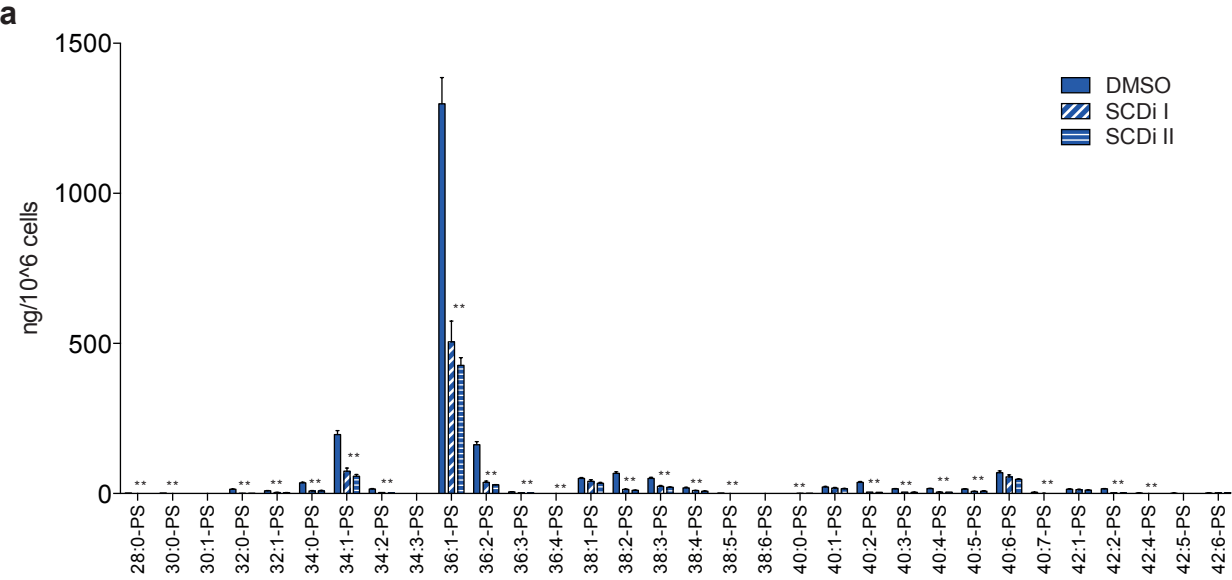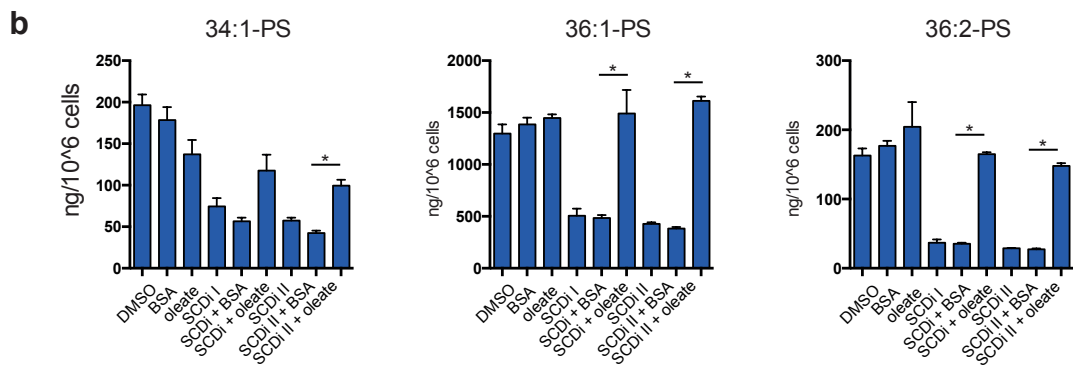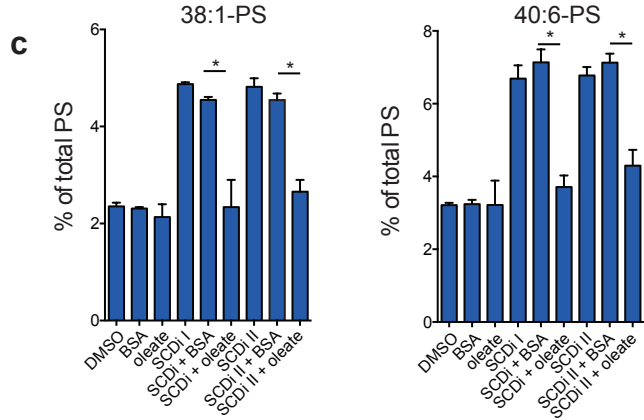

Supplement: Additional file 7: Figure S7. — Analysis of phosphatidylserine species in DU145 cells following SCD inhibition. (PDF 496 kb) [file 40170_2016_146_MOESM7_ESM.pdf]

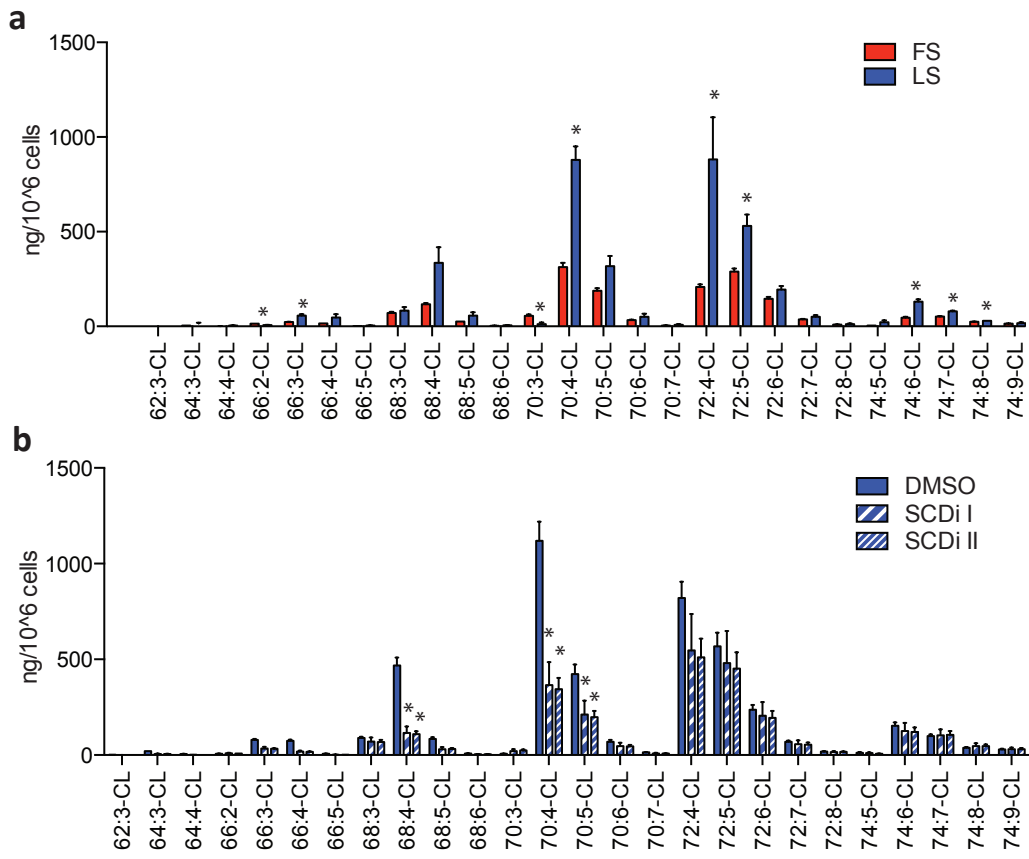

Supplement: Additional file 8: Figure S8. — Analysis of cardiolipin species in DU145 cells following SCD inhibition. (PDF 443 kb) [file 40170_2016_146_MOESM8_ESM.pdf]

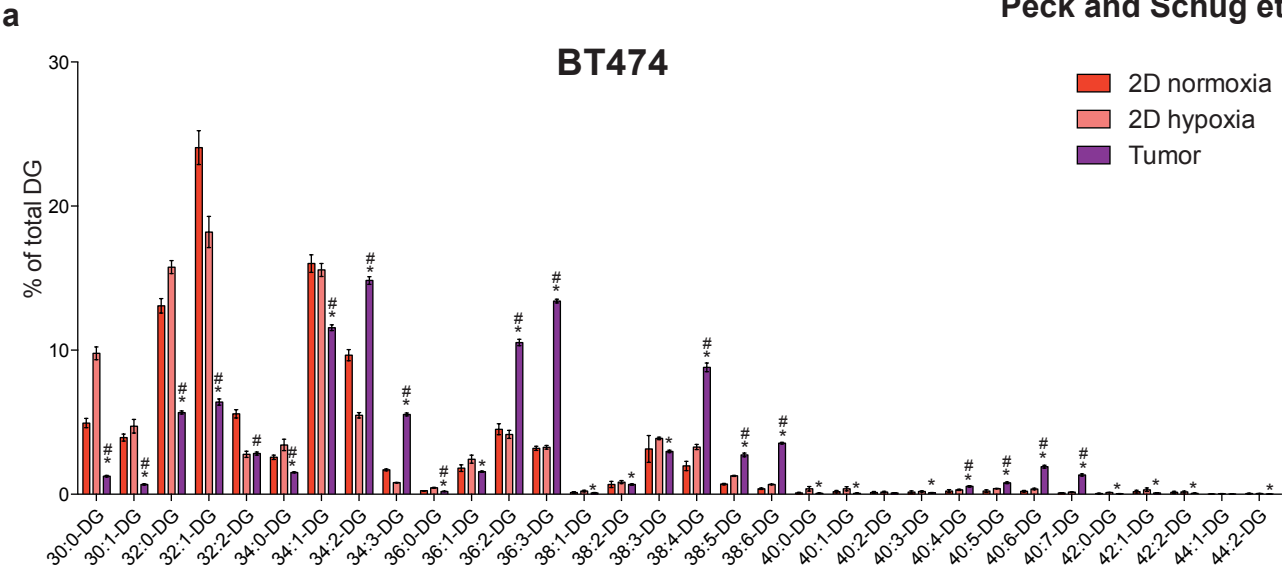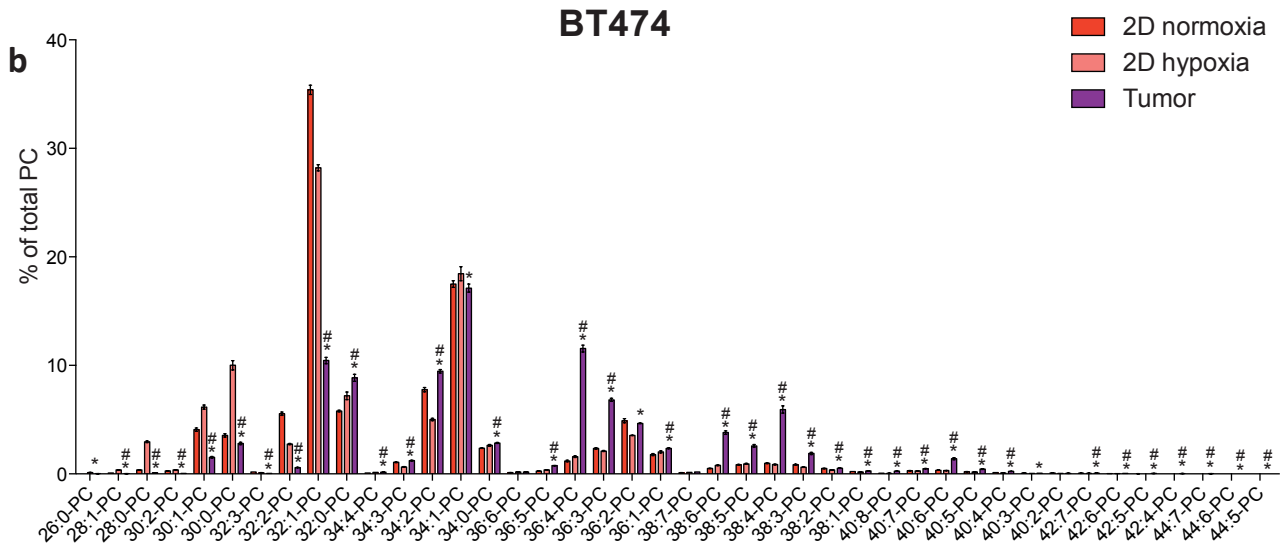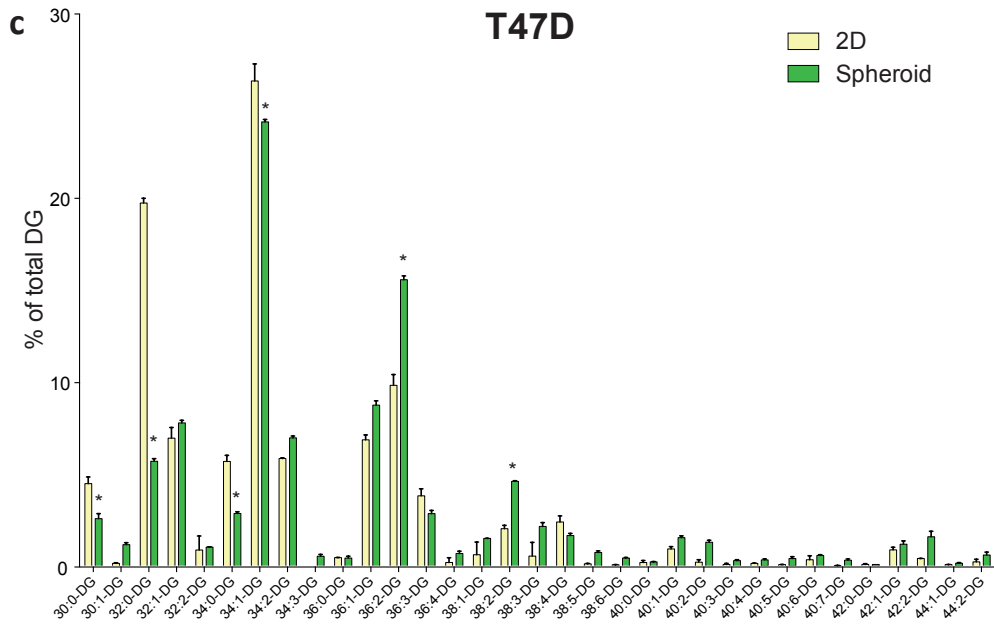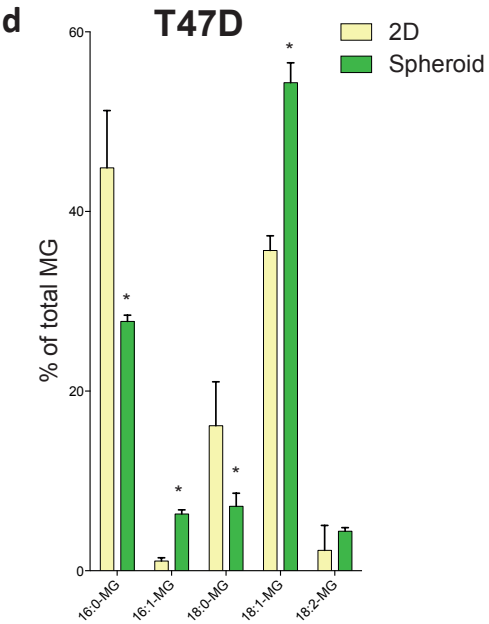

Supplement: Additional file 9: Figure S9. — Lipid species in tumour spheroids resemble those in orthotopic breast xenografts. (PDF 533 kb) [file 40170_2016_146_MOESM9_ESM.pdf]

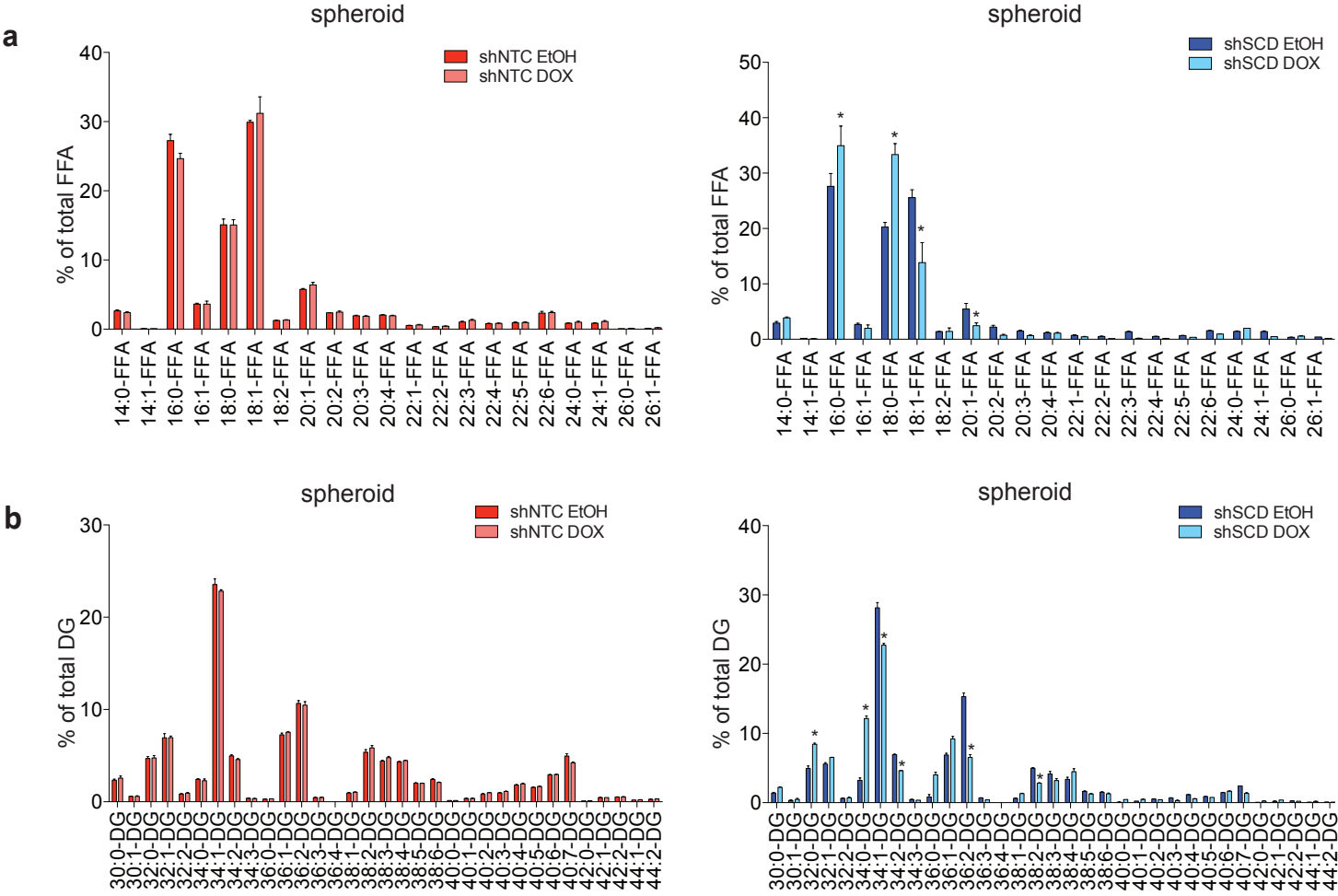

Supplement: Additional file 10: Figure S10. — Lipidomic analysis of DU145 tumour spheroids following SCD silencing. (PDF 497 kb) [file 40170_2016_146_MOESM10_ESM.pdf]
